# CCL20/CXCL5 Drives Crosstalk Between Anaplastic Thyroid Cancer Stem Cells and Tumor‐Associated Macrophages to Promote Tumor Progression

**DOI:** 10.1002/advs.202405399

**Published:** 2025-03-16

**Authors:** Qi Liu, Yan Wang, Mingyuan Song, Jiapeng Huang, Jinyuan Shi, Wei Sun, Xiaoyu Ji, Yuang Chang, Bing Ma, Ping Zhang, Yuanyuan Yan, Hao Zhang

**Affiliations:** ^1^ Department of Thyroid Surgery The First Hospital of China Medical University Shenyang 110801 P. R. China; ^2^ Department of Pharmacology School of Pharmacy China Medical University Shenyang 110122 P. R. China; ^3^ Department of Thyroid Surgery General Surgery Qilu Hospital of Shandong University Jinan 250012 P. R. China; ^4^ Department of Clinical Epidemiology and Evidence‐based Medicine The First Hospital of China Medical University Shenyang 110801 P. R. China

**Keywords:** anaplastic thyroid cancer, cancer stem cell, CCL20, crosstalk, CXCL5, tumor microenvironment

## Abstract

The dynamic interplay between tumor‐associated macrophages (TAMs) and anaplastic thyroid cancer (ATC) shapes the tumor microenvironment and facilitates ATC progression. However, the mechanisms of communication between TAMs and anaplastic thyroid cancer stem cells (ATCSCs) remain largely unelucidated. Integrative analyses of single‐cell RNA sequencing, cytokine/chemokine arrays, proteomics, and mRNA expression datasets are performed to reveal crosstalk between TAMs and ATCSCs and signaling pathways in ATCSCs. Subsequently, in vitro experiments are performed to validate the regulatory effects of key cytokines on ATCSC stemness. Last, xenogeneic orthotopic thyroid ATCSCs transplantation models are utilized to corroborate the regulatory effect of cytokines on stemness. CCL20 derived from THP‐1‐M2 activates the IRAK‐1/NF‐κB1/2 signaling pathway in ATCSCs, thereby positively regulating stemness characteristics and upregulating CXCL5 secretion. ATCSCs not only exhibit autocrine CXCL5 participation in the regulation of stemness but also demonstrate paracrine CXCL5 activity to recruit THP‐1‐Mφ and maintain the M2 phenotype. CCL20 and CXCL5 are involved in the crosstalk between TAMs and ATCSCs. The CCL20/CXCL5 axis plays a crucial role in the interaction between TAMs and ATCSCs, establishing a progressive tumor microenvironment.

## Graphical abstract

This study elucidates the crosstalk between tumor‐associated macrophages (TAMs) and anaplastic thyroid cancer stem cells (ATCSCs). The findings reveal that CCL20 from THP‐1‐M2 activates the IRAK‐1/NF‐κB1/2 pathway in ATCSCs, thereby enhancing CXCL5 secretion. Autocrine CXCL5 regulates tumor stemness, while its paracrine action recruits THP‐1‐Mφ and promotes M2 polarization, thus underscoring the critical role of the CCL20/CXCL5 axis in the progressive tumor microenvironment.

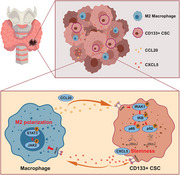

## 1 Introduction

Despite the generally favorable prognosis of thyroid cancer, anaplastic thyroid cancer (ATC) is rare and highly lethal.^[^ ^1^ ^]^ ATC typically manifests as a rapidly growing tumor with invasive neck masses. ≈50% of patients present with distant metastases at the time of initial diagnosis,^[^ ^2^, ^3^ ^]^ resulting in a median survival of ≈5 months and an overall 1‐year survival rate of 20%.^[^ ^4^ ^]^ Conventional therapeutic approaches, including surgical intervention and radiotherapy, have demonstrated limited efficacy in enhancing patient survival rates.^[^ ^5^ ^]^ In recent years, cancer stem cells (CSCs) have garnered significant attention due to their association with tumor progression, metastasis, recurrence, and treatment resistance.^[^ ^6^, ^7^ ^]^ Recent studies indicate that the activity of CSCs in ATC may play a crucial role in tumor recurrence and therapeutic resistance.^[^ ^8^ ^]^ Multiple signaling pathways, including MEK, JAK, and NF‐κB, are involved in the regulation of stemness in ATC.^[^ ^6^, ^8^, ^9^, ^10^ ^]^ ATC exhibits a higher proportion of CSCs compared to other types of thyroid cancer,^[^ ^11^ ^]^ which is crucial for poor prognosis; however, the underlying mechanism remains unclear.

Tumor‐associated macrophages (TAMs) constitute one of the primary components of the tumor microenvironment (TME) and fulfill diverse functions in tumor progression.^[^ ^12^ ^]^ In a variety of cancers, crosstalk exists between TAMs and tumor cells, which modulates malignant biological processes, including drug resistance, recurrence, and metastasis. For instance, the crosstalk between TAMs and colon cancer cells promotes colon cancer metastasis.^[^ ^13^ ^]^ Similarly, crosstalk between TAMs and hepatocellular carcinoma cells promotes the progression of hepatocellular carcinoma.^[^ ^14^ ^]^ Recent studies have reported crosstalk between ATC and TAMs.^[^ ^15^ ^]^ However, it remains uncertain whether such crosstalk also occurs between TAMs and anaplastic thyroid cancer stem cells (ATCSCs) in ATC, as well as whether this potential crosstalk serves as a primary driver of the elevated proportion of CSCs. The relationship between ATCSCs and TAMs remains incompletely elucidated. Although there are multiple reports on the crosstalk between TAMs and cancer cells, there is still a lack of consensus regarding the initial driving role in activating this crosstalk. For instance, TAMs can activate STAT3 in hepatocellular carcinoma cells through the secretion of IL‐6, inducing TAMs to release additional cytokines and form a positive loop, thereby maintaining the self‐renewal of hepatocellular carcinoma stem cells.^[^ ^16^ ^]^ TAMs have been reported to activate EphA4 through the secretion of ephrin, leading to the upregulation of cytokines IL‐1, IL‐6, and IL‐8, consequently promoting breast cancer progression.^[^ ^17^, ^18^ ^]^ Cancer cells can also initiate crosstalk. For example, cytokines secreted by CSCs participate in the establishment of a specific immunosuppressive microenvironment,^[^ ^19^, ^20^ ^]^ promoting tumor stemness.

Macrophages secrete diverse cytokines in distinct polarization states, and the M2 phenotype secretes various CC chemokine families.^[^ ^21^ ^]^ CCL20 was initially identified as a factor implicated in the etiology and progression of some autoimmune diseases, including psoriasis, arthritis, and inflammatory bowel disease.^[^ ^22^, ^23^ ^]^ CCL20 is a chemokine that exclusively activates the receptor CCR6.^[^ ^24^ ^]^ The role of CCL20/CCR6 in the progression of the tumor microenvironment is being progressively elucidated.^[^ ^25^ ^]^ CXCL5 is a small‐molecule chemokine that activates its specific receptor CXCR2 to induce immune cell migration and trigger inflammatory responses.^[^ ^26^ ^]^ CXCL5/CXCR2 contributes to the recruitment of immune cells and promotes angiogenesis.^[^ ^27^, ^28^ ^]^ However, the roles of CCL20, CXCL5, and their receptors in the TME remain largely unelucidated.

In this study, we confirmed the crosstalk between TAMs and ATCSCs, simulated alterations in cytokines in the TME, and identified the critical cytokines that modulate the stemness of ATCSCs. We constructed TAMs and ATCSCs models and co‐cultured them to investigate the potential crosstalk between them. Our findings found that CCL20 revealed that CCL20, which is predominantly secreted by THP‐1‐M2 macrophages, induces upregulation of CXCL5 secretion in ATCSCs. CXCL5 exhibits a dual function in regulating ATCSC stemness and inducing polarization of THP‐1‐Mφ toward the M2 phenotype. We further validated the role of crosstalk utilizing a xenogeneic orthotopic thyroid ATCSCs transplantation model that simulates the progressive TME. Mechanistically, CCL20 secreted by THP‐1‐M2 macrophages acts as an initial driver that activates CCR6 and upregulates IRAK1 phosphorylation in ATCSCs. The phosphorylation cascade of IRAK1 results in the upregulation of phosphorylation of NF‐κB1/2 and enhances the transcription of CXCL5. Our study demonstrated that crosstalk between ATCSCs and TAMs in the TME establishes a positive loop through CCL20 and CXCL5. This research identified a novel potential therapeutic target for ATC.

## 2 Results

### 2.1 THP‐1‐M2 Macrophages and ATCSCs Established a Progressive Tumor Microenvironment

**Figure 1:**
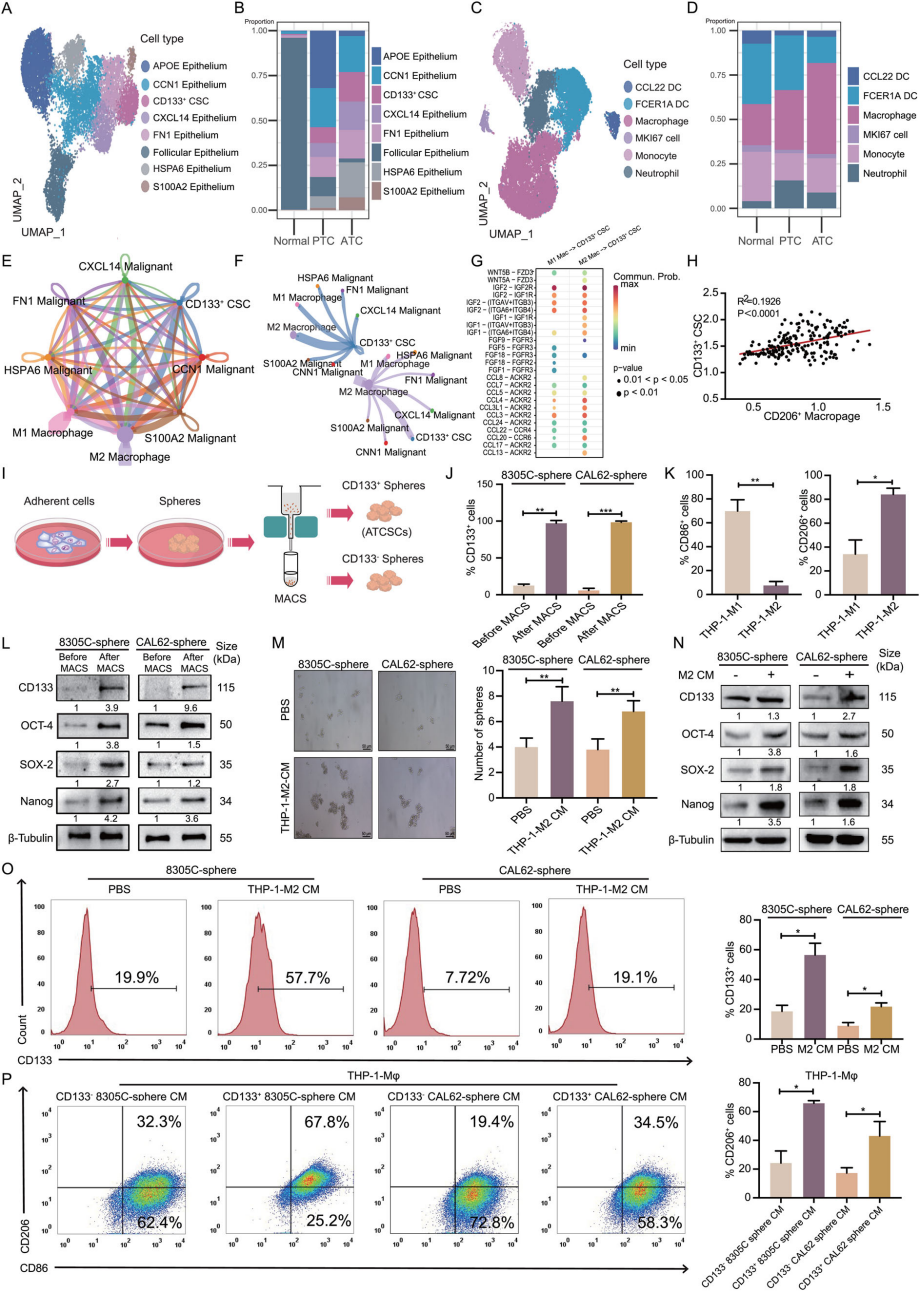
THP‐1‐M2 macrophages and ATCSCs establish an advanced tumor microenvironment. A) UMAP plot demonstrating 8 clusters based on unsupervised clustering for thyrocytes passing quality control. B) Bar plots indicate the proportion of 8 cell clusters in the normal thyroid tissue, PTC, and ATC groups. C) UMAP plot demonstrating 6 distinct clusters based on gene expression differences in myeloid cells. D) Bar plots indicate the proportion of 6 cell clusters in myeloid cells. E) Circle plots show the comparison of interaction quantity and interaction strength between CD206^+^ M2 macrophage and CD133^+^ CSC. F) Circle plots show the comparison of the interaction strength between CD206^+^ M2 macrophage and CD133^+^ CSC. G) Comparison of the differences in specific cell communication intensity between M1 and M2 macrophage with CD133^+^ CSC. H) The expression levels of CD206^+^ M2 macrophages were positively correlated with CD133^+^ CSC. I) Illustration of CD133^+^ ATCSCs induced by ATC cell lines sorted by magnet‐activated cell sorting. J) Flow cytometry analysis of changes in CD133 positive rates before and after magnetic‐activated cell sorting of spheres (*n* = 3). K) Flow cytometry analysis of changes in CD206 or CD86 positive rates of THP‐1‐M1 and THP‐1‐M2 (*n* = 3). L) The expression levels of CD133, OCT‐4, SOX‐2, and Nanog were detected in the spheres before and after magnetic‐activated cell sorting (*n* = 3). M) Self‐renewal ability of spheres was detected in co‐culture with or without THP‐1‐M2 CM (*n* = 3). N) The expression levels of CD133, OCT‐4, SOX‐2, and Nanog were detected in the co‐culture with or without THP‐1‐M2 CM (*n* = 3). O) Flow cytometry analysis of changes in CD133 positive rates of spheres in co‐culture with or without THP‐1‐M2 CM (*n* = 3). P) Flow cytometry analysis of changes in CD206 positive rates of THP‐1‐Mφ in co‐culture with CD133^+^ spheres or CD133^−^ sphere CM (*n* = 3). *p < 0.05, **p < 0.01, ***p < 0.001; Student's *t*‐test, Pearson correlation analysis, Error bars, mean ± SD.

To investigate the alterations in the TME of ATC at the single‐cell level, we performed single‐cell RNA sequencing analysis of Gene Expression Omnibus (GEO) datasets (GSE 148673, GSE193581, and GSE 232237) comprising 6 normal thyroid tissues, 14 papillary thyroid cancer (PTC) samples, and 20 ATC samples. Following the elimination of batch effects, UMAP visualization and clustering of 142 802 high‐quality cells revealed 9 distinct cell types, including B cells, endothelial cells, epithelial cells (encompassing cancer cells), and macrophages, among others (Figure S1A, Supporting Information). Subsequently, through the implementation of high‐precision dimensionality reduction and clustering techniques on epithelial cells, we identified 8 distinct epithelial cell clusters. For thyroid cancer, stemness markers such as CD133, ALDH‐1, OCT‐4, and Nanog are widely used to evaluate tumor stemness, with CD133 being the most recognized as a cell membrane marker.^[^ ^29^ ^]^ Through the application of the above‐mentioned stemness markers for stemness scoring on these eight cell clusters, we identified a subgroup of cells with markedly elevated stemness (Figure S1B, Supporting Information). Each cell type was characterized using typical markers and visualized utilizing UMAP (**Figure** **1**A). By integrating tissue differentiation scoring (TDS), normal follicular cells were distinguished from other malignant epithelial cells with other dedifferentiated malignant epithelial cells (Figure S1C, Supporting Information). CD133 has been utilized as a CSC marker to identify ATCSCs.^[^ ^29^ ^]^ The composition ratios of these 8 cell types exhibited significant differences across the different samples, with a higher proportion of CD133^+^ CSC in ATC compared to normal thyroid tissues and PTC (Figure 1B). To investigate alterations in immune cells within the TME, we performed dimensionality reduction clustering on myeloid cells (Figure 1C), which revealed a significant increase in the proportion of macrophages in ATC samples (Figure 1D). Furthermore, cell communication analysis revealed a potential interaction between M2 macrophages and CD133^+^ CSC (Figure 1E,F). To further investigate the correlation between M2 macrophages and CD133^+^ CSC, we conducted a specific receptor‐ligand analysis utilizing the CellChat database, thereby identifying several receptor‐ligand pairs that may be involved in the interaction between M2 macrophages and CD133^+^ CSC (Figure 1G). Furthermore, by mapping M2 macrophages and CD133^+^ CSC to the GEO database, we observed a correlation between their infiltration ratios (Figure 1H).

To investigate the potential crosstalk between TAMs and ATCSCs, we established TAM and ATCSC models. Initially, we generated spheres from two ATC cell lines, 8305C (BRAFV600E) and CAL62 (wild‐type), utilizing a serum‐free culture methodology. CD133^+^ spheres were subsequently enriched through sequential CD133^+^ magnetic‐activated cell sorting (MACS) until proportion of the CD133^+^ cells exceeded 90% (Figure 1I). This CD133^+^ spheroid population was regarded as ATCSC (Figure 1J; Figure S1D, Supporting Information). Stemness‐related markers, including SOX‐2, OCT‐4, and Nanog, were significantly elevated in CD133^+^ spheres (ATCSC) compared to those in CD133^−^ spheres (Figure 1L). Subsequently, a TAMs model was constructed by inducing THP‐1‐Mφ into THP‐1‐M1/2 macrophages through the addition of various cytokines. CD86 and CD206 are surface markers of THP‐1‐M1 and THP‐1‐M2 macrophages, respectively.^[^ ^30^, ^31^ ^]^ The polarization status of THP‐1‐M1/2 macrophages was verified by detecting differences in the positive rates of CD206 and CD86 using flow cytometry (Figure 1K; Figure S1E, Supporting Information). Two models were established based on these findings.

We speculated that the crosstalk between TAMs and ATCSCs is mediated by cytokines or chemokines. Consequently, we co‐cultivated THP‐1‐Mφ macrophages with conditioned medium (CM) from ATCSCs and co‐cultivated ATCSCs with CM from THP‐1‐M2 macrophages. The CM of THP‐1‐M2 macrophages increased the sphere formation rate of ATCSCs (Figure 1M) and upregulated the expression of stemness‐related markers (Figure 1N). In addition, flow cytometry analysis revealed an increase in the percentage of CD133^+^ spheres (Figure 1O). These findings demonstrate that the CM of THP‐1‐M2 macrophages positively regulates the stemness of ATCSCs. Subsequently, to verify the polarizing regulatory effect of ATCSC CM on THP‐1‐Mφ macrophages, we co‐cultured THP‐1‐Mφ macrophages with ATCSC CM following consecutive CD133^+^ magnet‐activated cell sorting. The CM of ATCSCs was co‐cultured with THP‐1‐Mφ macrophages, while the CM of CD133^−^ spheres were also co‐cultured as a reference. The CM of ATCSCs significantly promoted the polarization of THP‐1‐Mφ macrophages toward the M2 phenotype, as evidenced by the upregulated expression of CD163, CCL22, and IL‐10 and downregulated expression of IL‐1β and TNF‐α, which are genes associated with M1 polarization (Figure S1F, Supporting Information). Flow cytometry analysis demonstrated that CM of CD133^−^ spheres also induced polarization of THP‐1‐Mϕ macrophages toward an M2 phenotype, and the CM of ATCSCs exhibited a greater ability to induce M2 polarization (Figure 1P). Collectively, we established that CM‐mediated crosstalk exists between TAMs and ATCSCs, contributing to the development of a progressive TME and enhancing tumor stemness.

### 2.2 The CCL20/CXCL5 Axis is Critical for TAM and ATCSC Construction in a Progressive Tumor Microenvironment

**Figure 2:**
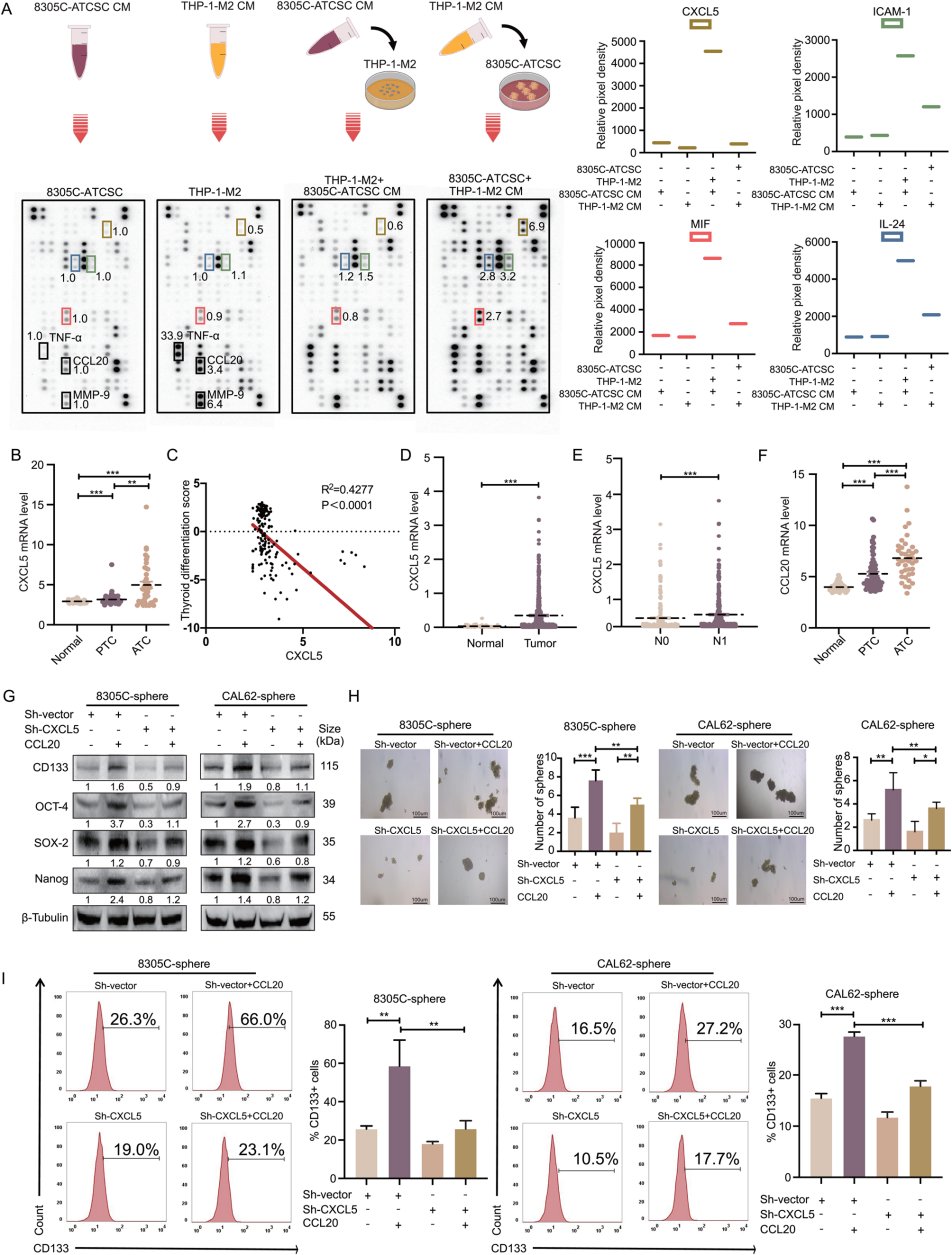
The CCL20/CXCL5 axis is critical for TAM and ATCSC construction in an advanced tumor microenvironment. A) Representative images of chemokine array analysis of 8305C‐ATCSC and THP‐1‐M2 and their co‐culture. B) The mRNA levels of CXCL5 were compared in normal thyroid tissue, PCT, and ATC from GEO datasets. C) Correlation analysis of CXCL5 mRNA expression and thyroid differentiation score in human thyroid cancers from GEO datasets. D) The mRNA levels of CXCL5 were compared between normal thyroid tissue and thyroid cancer from TCGA datasets. E) The mRNA levels of CXCL5 were compared in N0 and N1 stages from TCGA datasets. F) The mRNA levels of CCL20 were compared in normal thyroid tissue, PCT, and ATC from GEO datasets. G) The expression levels of CD133, OCT‐4, SOX‐2, and Nanog were detected in spheres with or without CCL20 and/or CXCL5 (*n* = 3). H) Self‐renewal ability of spheres was detected in co‐culture with or without CCL20 and/or CXCL5 (*n* = 3). I) Flow cytometry analysis of changes in CD133 positive rates of spheres in co‐culture with or without CCL20 and/or CXCL5 (*n* = 3). *p < 0.05, **p < 0.01, ***p < 0.001, ns, no significance; Mann–Whitney U analysis, Pearson correlation analysis, Two‐way ANOVA test, Error bars, mean ± SD.

Cytokines derived from CM are involved in the crosstalk between THP‐1‐M2 macrophages and ATCSCs. To investigate the specific cytokines involved in this crosstalk, we conducted an analysis of cytokine or chemokine changes in the CM of 8305C‐ATCSCs, THP‐1‐M2 macrophages, 8305C‐ATCSCs co‐cultured with the CM of THP‐1‐M2 macrophages, and THP‐1‐M2 macrophages co‐cultured with the CM of 8305C‐ATCSCs using a cytokine/chemokine array (**Figure** **2**A). Our investigation demonstrated that the secretion of several chemokines was altered after 8305C‐ATCSCs were co‐cultured with the CM of THP‐1‐M2 macrophages, including CXCL5, MIF, ICAM‐1, and IL‐24, which exhibited the most substantial changes (Figure 2A; Table S1, Supporting Information).

To identify the key chemokines involved in this crosstalk, we analyzed the GEO datasets (GSE33630, GSE 29265, and GSE76039) to determine their alterations in different types of thyroid cancer. The datasets comprised 40 ATC, 69 PTC, and 65 normal thyroid tissue samples. Among the four candidate cytokines or chemokines, only CXCL5 expression exhibited significantly higher levels in ATC tissues compared to PTC and normal thyroid tissues (Figure 2B). However, MIF, ICAM‐1, and IL‐24 levels did not demonstrate significant differences (Figure S2A, Supporting Information). Of particular importance, we assessed the correlation between CXCL5 and the thyroid differentiation score, revealing a significant negative correlation (Figure 2C). Moreover, we conducted an analysis of CXCL5 and clinically relevant parameters utilizing the TCGA database. The results demonstrated that CXCL5 expression exhibited statistically significant differences between thyroid cancer and normal thyroid tissue and displayed positive correlations with T stage, N stage, and AJCC stage (Figure 2D,E; Figure S2B, Supporting Information).

To investigate which cytokines derived from the CM of THP‐1‐M2 macrophages could induce the upregulation of CXCL5 secretion from 8305C‐ATCSCs, we selected cytokines that were highly secreted in THP‐1‐M2 macrophages and minimally or not secreted in 8305C‐ATCSCs, including TNF‐α, MMP‐9, and CCL20 (Figure 2A; Figure S2C, Supporting Information). Subsequently, we analyzed the mRNA expression of TNF‐α, MMP‐9, and CCL20 utilizing the GEO datasets. Our findings indicated that the expression of CCL20 and MMP‐9 in ATC was elevated compared to that in PTC and normal thyroid tissues, whereas no significant difference was observed in TNF‐α levels (Figure 2F; Figure S2D, Supporting Information). Additionally, we validated the protein expression levels of CCL20 in THP‐1‐Mφ, THP‐1‐M1, and THP‐1‐M2 macrophages. Our findings revealed that THP‐1‐M2 macrophages exhibited elevated levels of CCL20 secretion compared to THP‐1‐Mφ and THP‐1‐M1 macrophages (Figure S2E, Supporting Information). The STRING database was employed for protein‐protein interaction analysis, encompassing both physical and functional associations.^[^ ^32^ ^]^ We hypothesized a potential correlation between TNF‐α, MMP‐9, CCL20, and CXCL5. Our findings indicated that this association was observed exclusively between CCL20 and CXCL5 (Figure S2F, Supporting Information). Notably, we did not observe significant alterations in CXCL5 levels in THP‐1‐M2 macrophages co‐cultured with CM of 8305C‐ ATCSCs suggesting that 8305C‐ATCSCs derived CXCL5 may be secreted in response to stimulation by THP‐1‐M2 macrophage‐derived CCL20 (Figure 2A). These findings demonstrate that the initial induction of crosstalk is mediated by TAM‐derived CCL20 rather than by ATCSCs.

To elucidate the regulatory effects of the three potential cytokines on CXCL5 secretion from 8305C‐ATCSCs, we investigated the gene expression and protein level alterations of CXCL5 by introducing recombinant human CCL20, MMP‐9, and TNF‐α to ATCSCs. CCL20 upregulated the secretion levels of CXCL5 in ATCSCs, whereas the effects of TNF‐α and MMP‐9 on ATCSCs were not statistically significant (Figure S2G, Supporting Information). Subsequently, to demonstrate that CCL20 derived from TAM upregulates the secretion of CXCL5 by ATCSC, which constitutes a critical axis in regulating the stemness characteristics of ATCSC, we analyzed the expression of stemness‐related proteins in ATCSC under different conditions. The expression of stemness‐related markers in ATCSC was observed to be upregulated following the addition of recombinant human CCL20. Conversely, the inhibition of CXCL5 in ATCSC resulted in the downregulation of these proteins. Notably, the addition of CCL20 under conditions of CXCL5 inhibition in ATCSC did not fully restore the expression of stemness‐related markers. (Figure 2G). Subsequently, spheroid assays and flow cytometry analysis were conducted under identical conditions, yielding comparable results (Figure 2H, I). To investigate whether CCL20 derived from THP‐1‐M2 macrophages was induced by CXCL5, we silenced CCL20 in THP‐1‐Mφ cells. Our findings revealed that following CCL20 silencing, despite a significant decrease in CCL20 secretion, CXCL5 retained its capacity to induce polarization toward the M2 phenotype. (Figure S2H; Figure S2I, Supporting Information).

### 2.3 CCL20 Upregulates IRAK1 and Activates NF‐κB1/2 Through a Phosphorylation Cascade to Promote CXCL5 Transcription

**Figure 3:**
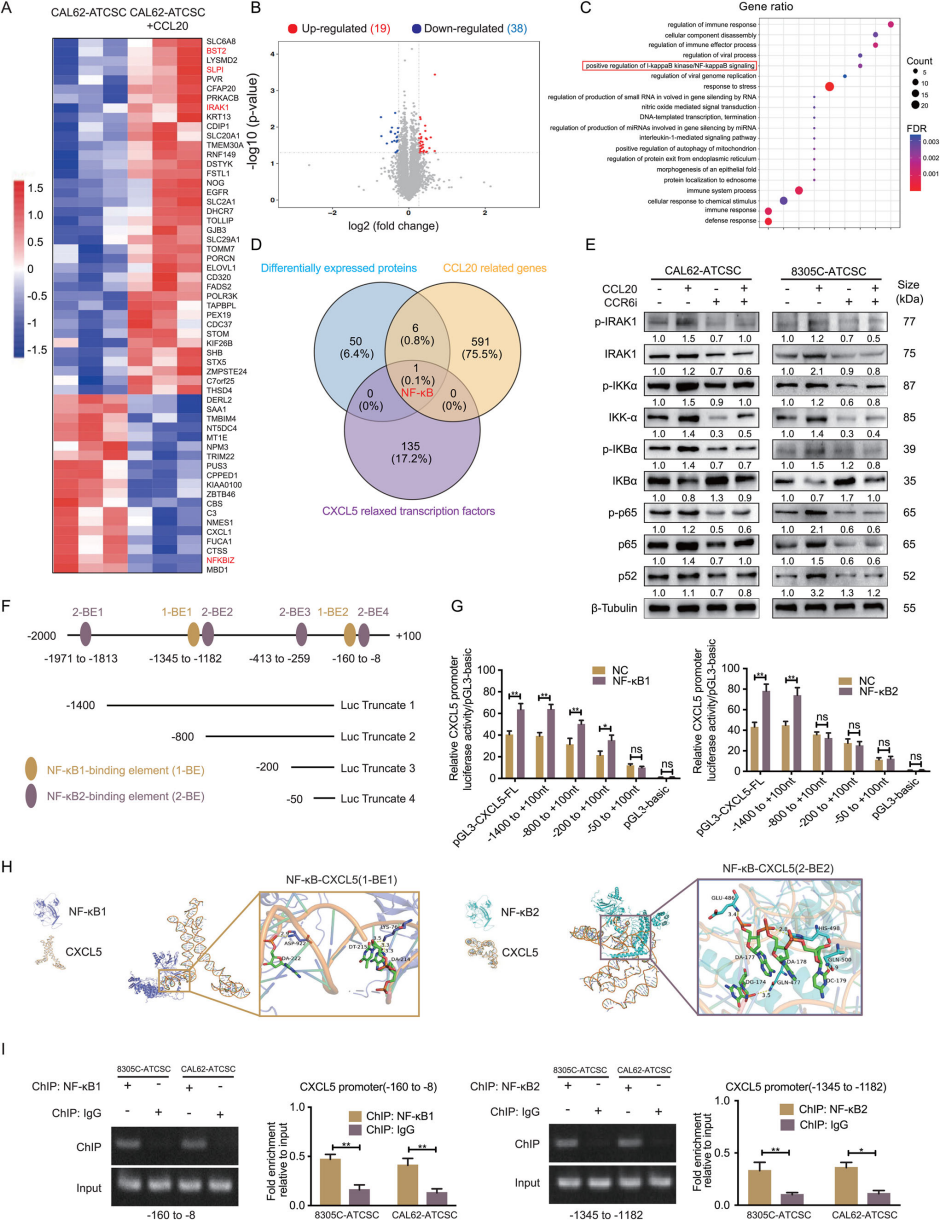
CCL20 upregulates IRAK1 and activates NF‐κB1/2 through a phosphorylation cascade to promote CXCL5 transcription. A) Differential protein levels in CAL62‐ATCSC with and without the addition of CCL20 analyzed by proteomics (*n* = 3). B) Upregulated versus downregulated proteins as revealed by proteomic analysis. C) Protein association network analysis and enrichment of potential signaling pathways. D) Intersection analysis of differential proteins, genes positively associated with CCL20, and potential transcription factors of CXCL5. E) The expression levels of IRAK‐1, IKK‐α, IKBα, P65, and P52, as well as the phosphorylation levels of p‐IRAK1, p‐IKKα, p‐IKBα, and p‐P65, were measured in ATCSC after the addition of CCL20 or CCR6 inhibitor (*n* = 3). F. Schematic diagram of potential binding elements of NF‐κB1/2 in the CXCL5 promoter region. G) Analysis of changes in luciferase activity of different truncated plasmids of NF‐κB1/2 using a dual luciferase reporter assay (*n* = 3). H. Molecular docking analysis of NF‐κB1/2 with CXCL5 specific binding elements. I. ChIP analysis of the binding ability between NF‐κB1/2 and the binding elements (*n* = 3). *p < 0.05, **p < 0.01, ns, no significance; Student's *t*‐test, Two‐way ANOVA test, Error bars, mean ± SD.

To investigate the positive regulation of stemness and CXCL5 expression by CCL20 in ATCSCs, we aimed to elucidate the potential mechanisms through proteomics. We performed a differential protein analysis comparing CAL62‐ATCSC treated with or without CCL20 (*n* = 3) to investigate the signaling pathway alterations that result in CXCL5 upregulation (**Figure** **3**A). CAL62‐ATCSC treated with CCL20 exhibited upregulation of 38 proteins and downregulation of 19 proteins (Figure 3B). Subsequent analysis revealed that the differentially expressed proteins BST2, SLPI, and IRAK1 were upregulated, whereas NFKBIZ, an inhibitor of NF‐κB, was downregulated (Figure 3A). Furthermore, KEGG enrichment analysis was performed for differentially expressed proteins, and stemness‐related pathway analysis revealed enrichment of the NF‐κB pathway (Figure 3C). Next, we intersected genes predicted to be positively correlated with CCL20 expression by GEPIA, potential transcription factors regulating CXCL5 by JASPAR and differentially expressed proteins by proteomics. Comparable results were obtained with the inclusion of NF‐κB (Figure 3D). Additionally, functional enrichment analysis of the proteins demonstrated significantly increased transcription and signal transduction (Figure S3A, Supporting Information). Previous studies have elucidated the role of NF‐κB in the regulation of stemness.^[^ ^33^, ^34^ ^]^ Based on these findings, we hypothesize that CCL20 activates the NF‐κB pathway to enhance CXCL5 transcription and synergistically interact with CXCL5 to modulate ATCSCs stemness.

Previous studies have demonstrated that IRAK1 phosphorylation activates the NF‐κB pathway.^[^ ^35^, ^36^ ^]^ Our proteomics results revealed that CCL20 could induce the upregulation of IRAK1 in ATCSCs. Consequently, we investigated alterations in IRAK1 and phosphorylated IRAK1 levels in ATCSCs following CCL20 stimulation. The results indicated that IRAK1 expression was significantly upregulated following CCL20 induction. Furthermore, we observed a phosphorylation cascade involving p‐IRAK1/p‐IKKα/p‐IκB in ATCSCs after CCL20 stimulation, and CCR6 inhibition suppressed this transcriptional effect (Figure 3E). In addition, our findings indicated that the expression of P‐52 and P‐65 was upregulated following CCL20 stimulation, which was accompanied by the upregulation of CXCL5, indicating concurrent activation of both the NF‐κB canonical and non‐canonical pathways. To elucidate the mechanism by which CCL20 activates NF‐κB to upregulate CXCL5 transcription, we constructed luciferase reporter plasmids containing the full‐length CXCL5 promoter (pGL3‐CXCL5‐FL) and four truncated CXCL5 promoter fragments (Figure 3F). The results of the dual‐luciferase reporter assay demonstrated that overexpression of either NF‐κB1 or NF‐κB2 enhanced the luciferase activity of pGL3‐CXCL5‐FL. Moreover, co‐expression of NF‐κB1 and NF‐κB2 exhibited an additive effect, further augmenting luciferase activity (Figure S3B, Supporting Information). However, in the truncated plasmids, truncation of NF‐κB1 binding site 4 of the NF‐κB2 binding site and truncations 2, 3, and 4 did not enhance luciferase activity (Figure 3G). To validate the association of NF‐κB1 and NF‐κB2 with these potential binding elements (BEs), we performed molecular docking of HDOCK to predict the binding activity between proteins and DNA. These findings indicated that both NF‐κB1 and NF‐κB2 were stably associated with specific BEs (Figure 3H).

To further validate the binding of NF‐κB1/2 to specific binding sites in the CXCL5 promoter region, we conducted chromatin immunoprecipitation (ChIP) assays. Our findings demonstrated that NF‐κB1 could bind directly to 1‐BE2 (‐86 bp), NF‐κB2 could bind directly to 2‐BE2 (‐1276bp), and no additional binding was observed in the other BEs. (Figure 3I; Figure S3C, Supporting Information).

Next, mutant plasmids containing 1‐BE2 and 2‐BE2 sequences were constructed in pGL3‐CXCL5‐FL to further validate the BEs of NF‐κB1 and NF‐κB2. Mutations in 1‐BE2 and 2‐BE2 significantly reduced the binding of NF‐κB1 and NF‐κB2 to the CXCL5 promoter (Figure S3D, Supporting Information). ChIP results demonstrated that overexpression of CCL20 enhanced the binding efficiency of NF‐κB1 and NF‐κB2 to their respective BEs in CAL62‐ATCSC and 8305C‐ATCSC. Conversely, CCR6 inhibition reversed this effect (Figure S3E, Supporting Information). Following the inhibition of CCR6 in ATCSC, the addition of rhCCL20 did not result in an increase in CXCL5 secretion (Figure S3F, Supporting Information). In conclusion, CCL20/CCR6 initiated the phosphorylation cascade of IRAK1, leading to the activation of NF‐κB1/2. This activation facilitates the transcription of CXCL5 in ATCSCs by binding to a specific CXCL5 promoter region.

### 2.4 CXCL5 Derived from ATCSCs Recruits THP‐1‐Mφ Macrophages and Induces Polarization Toward the M2 Phenotype

**Figure 4:**
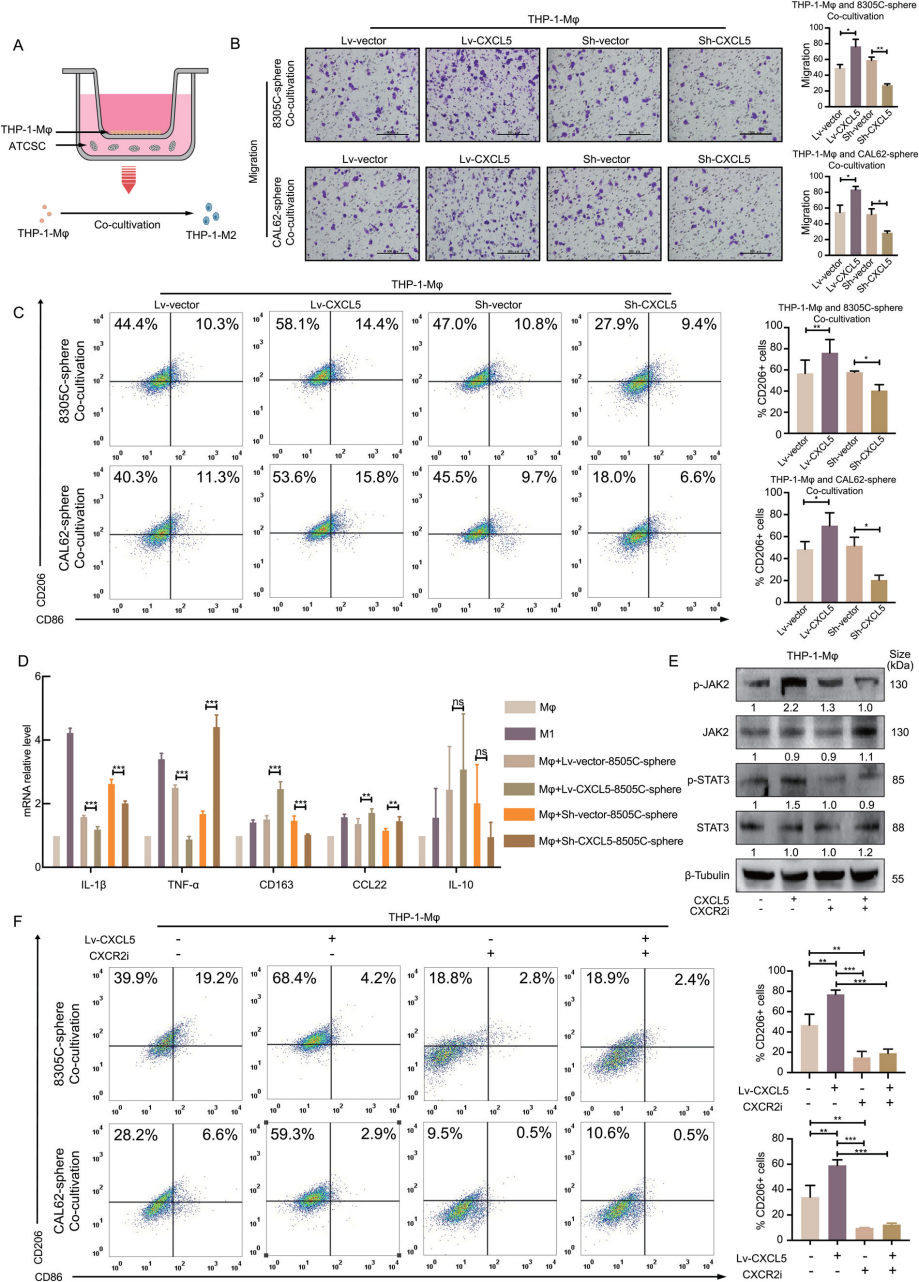
CXCL5 derived from ATCSCs recruits THP‐1‐Mφ macrophages and induces polarization toward the M2 phenotype. A) Illustration of indirect co‐culture between THP‐1‐Mφ and ATCSC. B) Analysis of migration ability of THP‐1‐Mφ after indirect co‐culture with 8305C‐ATCSC or CAL62‐ATCSC overexpressing or silencing of CXCL5 (*n* = 3). C) Flow cytometry analysis of THP‐1‐Mφ after indirect co‐culture with 8305C‐ATCSC or CAL62‐ATCSC overexpressing or silencing CXCL5. D) The mRNA levels of IL‐1β, TNF‐α, CD163, CCL22, and IL‐10 were measured in THP‐1‐Mφ after indirect co‐culture with 8305C‐ATCSC overexpressing or silencing CXCL5. E) The expression levels of JAK2 and STAT3, as well as the phosphorylation levels of p‐JAK2 and p‐STAT3, were measured in ATCSC after the addition of CXCL5 or CXCR2 inhibitor (*n* = 3). F) Flow cytometry was used to analyze the changes in CD206 positivity rate in THP‐1‐Mφ after the addition of CXCL5 or CXCR2 inhibitor (*n* = 3). *p < 0.05, **p < 0.01, ***p < 0.001; Student's *t*‐test, two‐way ANOVA test, Error bars, mean ± SD.

The role of ATCSC‐derived CXCL5 in macrophage recruitment and polarization in the TME remains inadequately elucidated. Consequently, we investigated the recruitment and polarization effects of CXCL5 on macrophages in the TME utilizing a co‐culture system. This system comprised co‐culturing of THP‐1‐Mφ macrophages with Lv‐CXCL5‐ATCSCs. To further substantiate the recruitment effect of ATCSCs on THP‐1‐Mφ macrophages, we performed a Transwell assay. Lv‐CXCL5‐ATCSCs were positioned in the lower chamber, and THP‐1‐Mφ macrophages were placed in the upper chamber of the co‐culture system (**Figure** **4**A). These results demonstrated that Lv‐CXCL5‐ATCSCs exhibited a more pronounced effect on the recruitment of THP‐1‐Mφ macrophages (Figure 4B). More importantly, flow cytometry analysis revealed that CM of Lv‐CXCL5‐ATCSCs significantly increased the proportion of CD206^+^ TAMs, indicating polarization toward the M2 subtype, compared to Sh‐CXCL5‐ATCSCs (Figure 4C).

Furthermore, our investigation revealed that in the co‐culture system, Lv‐CXCL5‐ATCSCs induced the polarization of THP‐1‐Mφ macrophages toward the M2 phenotype. We observed an upregulation of M2‐related macrophage genes, whereas the expression of M1‐related macrophage genes exhibited no significant change or even downregulation, as detected by co‐culturing THP‐1‐Mφ macrophages with Lv‐CXCL5‐ATCSCs. However, the effect of Sh‐CXCL5‐ATCSCs on THP‐1‐Mφ macrophages was not significant (Figure 4D).

Previous studies have demonstrated that CXCR2 activation can lead to activation of the JAK2‐STAT3 pathway.^[^ ^37^ ^]^ Our research also indicated that CXCL5 upregulated the phosphorylation of JAK2 (p‐JAK2) and STAT3 (p‐STAT3) in THP‐1‐Mφ macrophages. Consequently, CXCL5 secreted by ATCSCs activated JAK2‐STAT3 signaling in THP‐1‐Mφ macrophages and supported the maintenance of the M2 phenotype (Figure 4E). Subsequently, we silenced CXCR2 in THP‐1‐Mφ and co‐cultured them with the CM of Lv‐CXCL5‐ATCSC. Our findings revealed that after silencing CXCR2 in THP‐1‐Mφ, the CM of Lv‐CXCL5‐ATCSC no longer induced an increase in CD206 expression (Figure 4F). In conclusion, our research has demonstrated that the secretion of CXCL5 by ATCSCs not only recruits THP‐1‐Mφ macrophages but also induces their polarization toward an M2 phenotype.

### 2.5 CXCL5 is Essential for Promoting the Stemness Characteristics of ATCSCs

**Figure 5:**
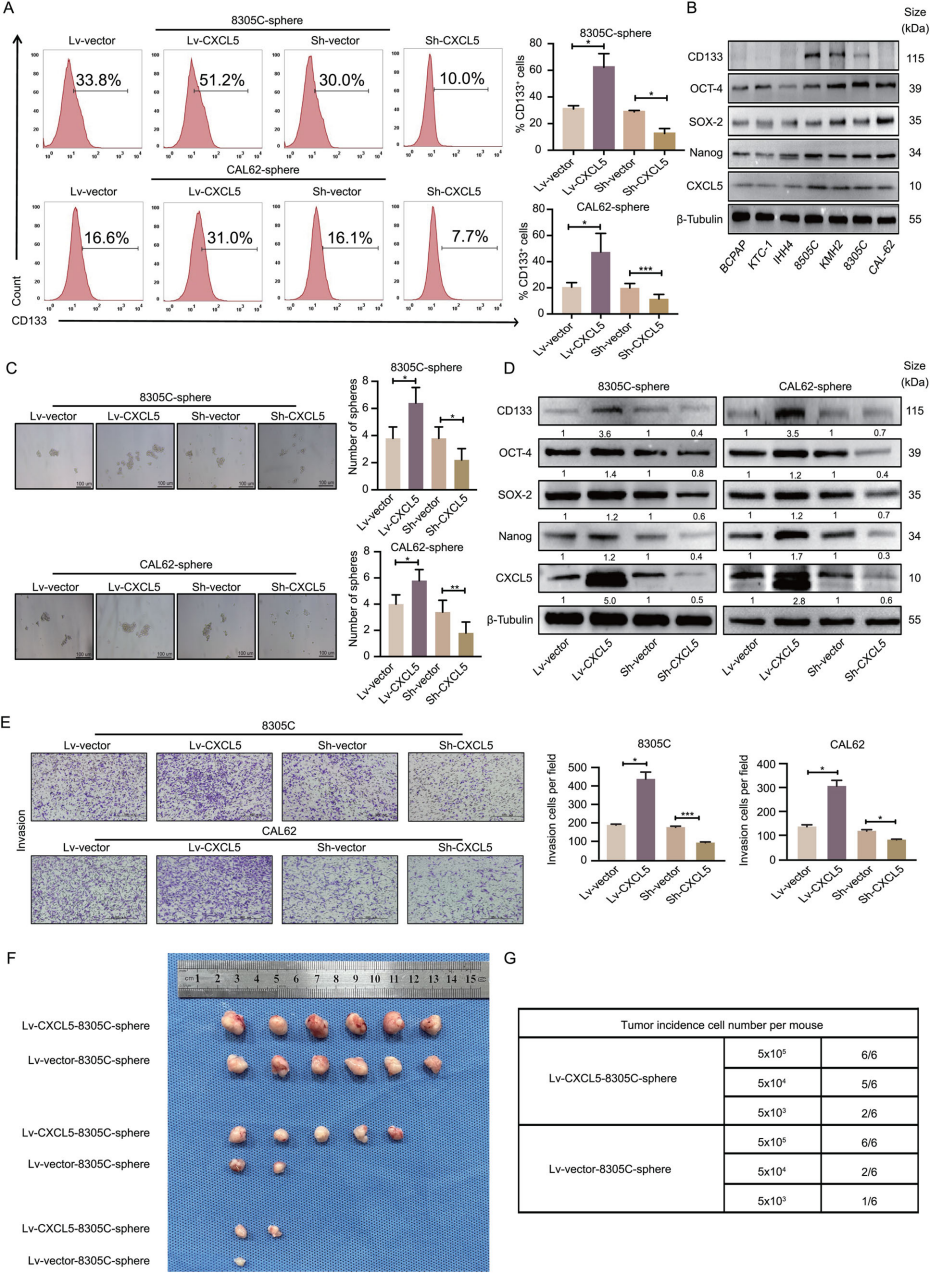
CXCL5 is essential for promoting the stemness characteristics of ATCSCs. A) Flow cytometry was used to analyze the changes in CD133 positivity rate in Lv‐CXCL5‐sphere and Sh‐CXCL5‐sphere (*n* = 3). B) The expression levels of CD133, OCT‐4, SOX‐2, Nanog, and CXCL5 were measured in three thyroid papillary carcinoma cell lines (BCPAP, KTC‐1, and IHH4) and four ATC cell lines (8305C, KMH2, 8305C, and CAL62). C) The self‐renewal ability of spheres was detected in Lv‐CXCL5‐sphere and Sh‐CXCL5‐sphere (*n* = 3). D) The expression levels of CD133, OCT‐4, SOX‐2, Nanog, and CXCL5 were measured in Lv‐CXCL5‐sphere and Sh‐CXCL5‐sphere (*n* = 3). E) The invasion ability of 8305C and CAL62 with CXCL5 overexpression or silencing was analyzed using Transwell assays (*n* = 3). F,G) Changes in limiting dilution tumorigenesis rate and tumor size of 8305C‐sphere overexpressing or silencing CXCL5 in vivo (*n* = 6). *p < 0.05, ***p < 0.001; Student's *t*‐test, Error bars, mean ± SD. *p < 0.05, **p < 0.01, ***p < 0.001; Student's t test, Error bars, mean ± SD.

To analyze the role of CXCL5 in the regulation of ATCSC stemness, we successfully constructed lentiviral vectors to transfect ATCSCs. Flow cytometry was performed to investigate the correlation between CXCL5 and CD133 expression. Flow cytometry analysis revealed that the rate of CD133 positivity in Lv‐CXCL5‐ATCSCs was significantly higher than that in Lv‐vector‐ATCSCs. Conversely, when CXCL5 was silenced, the CD133 positive rate of ATCSC exhibited a significant decrease (**Figure** **5**A). ATC demonstrated increased stemness and aggressiveness compared to PTC.^[^ ^29^ ^]^ We hypothesized that CXCL5 independently regulates stemness. We investigated the potential correlation between CXCL5 and stemness markers in 3 PTC cell lines (BCPAP, KTC‐1, and IHH4) and 4 ATC cell lines (8505C, KMH2, 8305C, and CAL62). Our investigation revealed a positive correlation between CXCL5 expression and stemness markers CD133, OCT‐4, SOX‐2, and Nanog (Figure 5B). This finding indicates that CXCL5 is a crucial chemokine that regulates the stemness of ATCSCs. Following CD133^+^ magnet‐activated cell sorting, we observed increased secretion of CXCL5 in CD133^+^ 8305C‐spheres and CD133^+^ CAL62‐spheres compared to that in CD133^−^ 8305C‐spheres and CD133^−^ CAL62‐spheres (Figure S4A, Supporting Information). Subsequently, we conducted spheroid formation assays to investigate the regulatory role of CXCL5 in tumor stemness characteristics. Our findings revealed that Lv‐CXCL5‐ATCSCs exhibited a higher sphere formation rate than Lv‐vector‐ATCSCs, whereas the sphere formation rate of Sh‐CXCL5‐ATCSCs was lower than that of Sh‐vector‐ATCSCs (Figure 5C). These findings indicate that Lv‐CXCL5‐ATCSCs exhibit a higher self‐renewal capacity. Furthermore, Western blot analysis of stemness‐related markers revealed a significant increase in stemness‐related indicators in Lv‐CXCL5‐ATCSC; conversely, when CXCL5 was silenced, the expression of stemness‐related markers decreased (Figure 5D).

To exclude the influence of CXCL5 on the proliferation and invasion of parental ATC cells, in vitro experiments were performed. Neither overexpression nor silencing of CXCL5 significantly altered cell proliferation rates (Figure S4B, Supporting Information). Interestingly, ATC cells treated with rhCXCL5 exhibited significantly increased invasiveness and migration (Figure 5E; Figure S4C, Supporting Information). These findings revealed that CXCL5 had no significant effect on the proliferation of ATC cells but influenced their migration. To further verify the capacity of CXCL5 to regulate the stemness of ATCSCs and enhance their self‐renewal ability, we performed in vivo experiments using a mouse model. The results demonstrated that the Lv‐CXCL5‐ATCSCs group exhibited significantly higher tumor formation and tumor growth compared to the Lv‐vector‐ATCSC group (Figure 5F,G). Collectively, CXCL5 is essential for the positive regulation of ATCSCs stemness both in vivo and in vitro.

### 2.6 In Vivo and Clinical Evidence of CCL20/CXCL5 Axis in Progressive Tumor Microenvironment

**Figure 6:**
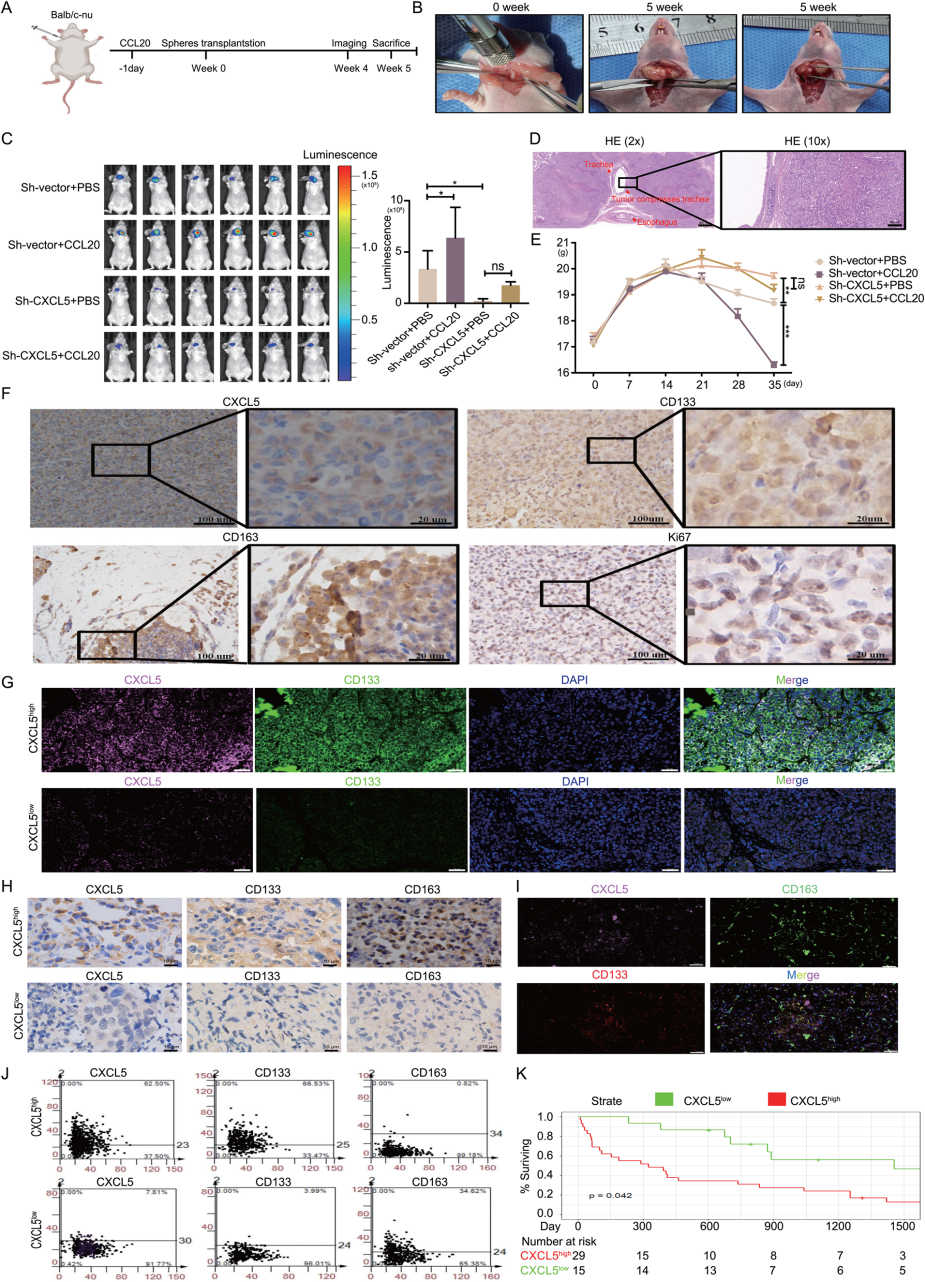
In vivo and clinical evidence of CCL20/CXCL5 axis in advanced tumor microenvironment. A) Illustration of the ATCSC orthotopic transplantation model. B) ATCSC transplantation in BALB/c‐nu mice and the invasion of tumors after 5 weeks. C) The in vivo images of mouse tumors with different treatments were detected using the imaging system (*n* = 6). D) HE staining of the thyroid orthotopic xenograft model in BALB/c‐nu mice. E) Differences in body weight changes over time between groups of BALB/c‐nu mice. F) Immunohistochemical analysis showing representative images of CXCL5, CD133, CD163, and Ki67 expression in the thyroid orthotopic xenograft model. G) Immunofluorescence co‐localization analysis showing representative images of CXCL5, CD133, CD163, and Ki67 expression in the thyroid orthotopic xenograft mode. H) Immunohistochemical analysis showing representative images of the correlation between CXCL5, CD133, and CD163 in human ATC tissues. I) Immunofluorescence co‐localization analysis showing representative images of CXCL5, CD133, CD163, and Ki67 expression in human ATC tissues. J) The correlation of CXCL5, CD133, and CD163 in human ATC tissues was analyzed by flow cytometry with panoramic immunohistochemistry. K) Survival analysis of ATC patients with CXCL5 expression using the Kaplan‐Meier curve. *p < 0.05, **p < 0.01, ***p < 0.001, ns, no significance; Two‐way ANOVA test, Error bars, mean ± SD.

We established a xenogeneic orthotopic thyroid ATCSCs transplantation model to simulate the invasion of ATC cells in vivo. This model facilitated a more accurate simulation of ATC invasion within a specific anatomical structure and the state of tumor growth in specific endocrine environments. The day prior to transplantation, intravenous injections of 100 ng rhCCL20 were administered. The next day, we utilized a Hamilton syringe (36G) to inject different groups of ATCSCs into the right thyroid of the mice (**Figure** **6**A,B). At week 4, in vivo, imaging of the models in each group was performed. Our findings demonstrated that tumor growth was accelerated in the Sh‐vector+CCL20 group, whereas it was decelerated in the Sh‐CXCL5+PBS and Sh‐CXCL5+CCL20 groups (Figure 6C). In the fifth week, the mice were euthanized and dissected to examine tumor growth in vivo. Tumors in the Sh‐vector+CCL20 group exhibited increased invasiveness, resulting in compression of the esophagus and trachea (Figure 6D). Changes in body weight and survival status in xenograft models. Models in the Sh‐vector+CCL20 group demonstrated significant weight loss and exhibited difficulty in food and water intake, indicating a cachexia‐like state. In contrast, the Sh‐CXCL5+PBS and Sh‐CXCL5+CCL20 groups exhibited no difficulty in food or water intake. Additionally, the models in the Sh‐vector+CCL20 group exhibited reduced body weight, whereas those in the Sh‐CXCL5+PBS and Sh‐CXCL5+CCL20 groups displayed no significant body weight loss (Figure 6E).

Subsequently, we performed immunohistochemistry (IHC) and immunofluorescence co‐localization analyses of the tumors. Notably, regions exhibiting elevated CXCL5 expression demonstrated a higher proportion of CD133^+^ ATCSCs, infiltration of CD163^+^ TAMs, and increased Ki67 expression (Figure 6F). Immunofluorescence co‐localization revealed that the highly secreted 8305C of ATCSC was positively correlated with CD133^+^ ATCSC (Figure 6G). In summary, our findings demonstrate that crosstalk between CCL20 and CXCL5 effectively promotes the self‐renewal of ATCSCs in xenograft models.

To assess the prognostic significance of CXCL5 in ATC patients, we obtained clinical data from 44 ATC patients to examine the potential correlation between CXCL5 expression and clinicopathological characteristics. IHC results indicated a positive correlation between CXCL5 expression and CD133^+^ ATCSCs, as well as CD163^+^ TAMs (Figure 6H). Subsequently, we performed immunofluorescence co‐localization of CXCL5, CD133, and CD163 in these pathological samples to elucidate the spatial relationship between CXCL5, ATCSCs, and TAMs. These findings demonstrated that regions with elevated CXCL5 expression exhibited a higher proportion of CD133^+^ ATCSCs and infiltration of CD163^+^ TAM. However, this phenomenon was not observed in CXCL5‐low samples (Figure 6I). We performed panoramic scanning of tissue sections to detect the positive rate in CXCL5‐high and CXCL5‐low samples and observed a similar enrichment of CD133^+^ ATCSCs and CD163^+^ TAMs in CXCL5‐high samples. However, this correlation was not observed in CXCL5‐low samples (Figure 6J). Collectively, CXCL5 secretion enhanced the proportion of CD133^+^ ATCSCs and recruited CD163^+^ TAMs to ATC tissues.

To investigate the correlation between CXCL5 and prognosis in patients with ATC, we analyzed the clinical data of 44 patients with ATC; the results are summarized in **Table** **1**. Our analysis revealed that ATC patients with high CXCL5 expression had a low probability of achieving R0 resection, suggesting that ATCs with high CXCL5 expression exhibit more aggressive invasiveness. We conducted survival analysis of CXCL5‐high and ‐low samples and plotted Kaplan‐Meier curves. The results indicated a poorer prognosis in the CXCL5‐high group compared to the CXCL5‐low group (Figure 6K). Therefore, these findings demonstrate that high CXCL5 expression is positively correlated with poor prognosis in ATC. Consequently, CXCL5 may serve as a potential prognostic indicator.

**Table 1:** Correlation analysis of CXCL5 expression levels with clinicopathological features in ATC patients (*n* = 44).

Parameters	*n*	CXCL5 expression level		
		High	Low	*p*
Sex				
Male	11	9	2	0.46
Female	33	21	12	
Age, years				
<55	9	5	4	0.61
≥55	35	25	10	
Tumor size				
≤4cm	7	4	3	0.66
>4cm	37	26	11	
Lymph node				
N0	19	11	8	0.62
N1	19	16	3	
Nx	6	3	3	
Metastasis				
M0	43	29	14	1
M1	1	1	0	
AJCC stage				
IVA	7	4	3	0.85
IVB	36	25	11	
IVC	1	1	0	
Surgical methods				
R0	32	18	14	0.02
R1/2	12	12	0	

## 3 Discussion

Currently, there is an urgent need to identify an effective treatment to inhibit the progression of ATC. CD133^+^ CSCs may be potential therapeutic targets.^[^ ^38^, ^39^ ^]^ We report for the first time that crosstalk between TAMs and ATCSCs, as well as chemokine‐mediated crosstalk, is involved in progressive TME. More importantly, we discovered that this crosstalk is driven by TAMs rather than tumor cells, which differs from previous reports.^[^ ^13^, ^40^ ^]^ Our study demonstrates that THP‐1‐M2 derived CCL20 induces CXCL5 secretion by ATCSCs. CXCL5 exhibits a dual role in crosstalk: autocrine regulation of ATCSCs stemness, paracrine recruitment of THP‐1‐Mφ, and induction of M2 polarization. Mechanistically, THP‐1‐M2 macrophage‐secreted CCL20 binds to CCR6 in ATCSCs and upregulates IRAK1 phosphorylation. This phosphorylation event initiates a cascade that ultimately activates both the canonical and non‐canonical NF‐κB pathways and enhances the transcription of CXCL5. Consequently, ATCSCs upregulate CXCL5 secretion, which recruits THP‐1‐M2 macrophages through paracrine signals to recruit TAMs and induce their polarization toward the M2 phenotype. This process establishes a positive loop that perpetuates the stemness of ATCSCs and facilitates the development of cancer stem cell niches within the progressive TME.

In 2009, Hernandez et al. confirmed that macrophages and tumor cells establish crosstalk between CSF‐1 and EGF.^[^ ^41^ ^]^ This crosstalk is considered to promote tumor progression; however, the role of CSCs in this crosstalk has not been elucidated. A variety of cytokines or chemokines may mediate this crosstalk. The infiltration has been extensively investigated for its clinical significance in predicting poor prognosis in various types of malignant tumors.^[^ ^13^, ^42^, ^43^ ^]^ TAMs are prevalent in ATC tissue samples.^[^ ^44^, ^45^ ^]^ The immune microenvironment facilitates ATC to evade immune surveillance, with cytokine interactions being the key mediators of communication between these microenvironments. TAMs have been identified as the primary source of CCL20 in metastatic primary melanoma.^[^ ^46^ ^]^ A single‐cell RNA‐sequencing study revealed that infiltrating TAMs in head and neck squamous cell carcinoma secrete higher levels of CCL20 in early‐stage tumors.^[^ ^47^ ^]^ Considering the crucial role of cytokines in the establishment of the TME between TAMs and ATCSCs, we identified and validated that CCL20 and CXCL5 promoted ATCSCs self‐renewal. Inhibition of cytokine‐mediated crosstalk represents a departure from conventional cancer therapy methods. This approach focuses on the “reprogramming” strategy of the TME and alters the survival environment of cancer cells.

In this crosstalk, a complex network of signals collectively maintains the stemness of ATCSCs. CCL20 secreted by THP‐1‐M2 can promote the activation of the NF‐κB signaling pathway, and the activation of NF‐κB positively regulates stemness.^[^ ^48^ ^]^ Recent studies have elucidated the multifunctionality of most transcription factors, as they play pivotal roles in transcriptional regulation and responses to various stimuli. Although the interaction between CCL20 and CXCL5 has not been fully elucidated, several studies have suggested a connection. These studies investigated the potential synergy in immune cell recruitment^[^ ^49^ ^]^ and the possible involvement of NF‐κB activation.^[^ ^50^ ^]^ Our findings demonstrated that the mechanism by which CCL20 upregulates CXCL5 expression involves the upregulation of IRAK1 levels in cells upon activation of CCR6 by CCL20, as determined by proteomic pathway enrichment analysis. IRAK1 and p‐IRAK1 can activate NF‐κB‐related pathways, resulting in increased transcriptional activity.^[^ ^35^, ^51^, ^52^ ^]^ Our study revealed that p‐IRAK1 activates both the canonical and non‐canonical NF‐κB pathways through a cascade of phosphorylation reactions. This dual signaling pathway activation is observed in multiple myeloma,^[^ ^53^ ^]^ potentially resulting from IKKα phosphorylation, which activates both canonical and non‐canonical pathways.^[^ ^54^ ^]^ Furthermore, CXCL5 secreted by ATCSCs positively regulates stemness. Based on our findings, we confirmed that CCL20 and CXCL5 cooperatively regulate the stemness characteristics of ATCSCs. We also verified the crucial role of CXCL5 in this crosstalk in xenograft models. Although the release of CXCL5 by ATCSCs was significantly increased upon CCL20 activation of CCR6, there was no significant alteration in its intracellular concentration, which also resulted in the absence of differential CXCL5 expression in proteomic analysis. This may be attributed to the fact that CXCL5 is primarily secreted extracellularly. In our study, although distant metastases were infrequently observed with the use of a live animal imaging system, this may be due to rapid tumor growth or unremarkable imaging of Luc‐labeled micro‐metastases. We obtained similar results in IHC validation of tissue samples from 44 patients with ATC. Both in vivo experiments and clinical data suggest that high CXCL5 expression in ATC is positively correlated with stemness and invasiveness, as well as a higher proportion of TAM infiltration. To our knowledge, this is the first report of chemokine‐mediated crosstalk between TAMs and ATCSCs in the mouse thyroid gland.

Numerous studies have reported that CXCL5 promotes cancer progression.^[^ ^55^, ^56^, ^57^ ^]^ However, the role of CXCL5 in the regulation of stemness in CSCs and its interaction with immune cells has not been thoroughly investigated. Current research has demonstrated the potential of targeting the CXCR2 receptor of CXCL5 to regulate immune escape in tumors. Inhibition of CXCR2 suppresses myeloid‐derived suppressor cell recruitment and enhances anti‐PD‐1 therapeutic efficacy in rhabdomyosarcoma.^[^ ^58^ ^]^ Additionally, previous studies have demonstrated that CXCL5 recruits neutrophils and other immune cells to the TME.^[^ ^59^, ^60^ ^]^ However, the effect of CXCL5, either independently or in combination with other cytokines, on polarization toward the M2 phenotype remains unexplored. Our findings indicated that CXCL5 recruits macrophages to the TME. Nevertheless, the observed macrophages could be in a transitional state, as we utilized the THP‐1 cell line to model macrophage polarization instead of studying phenotypic changes in TAMs in cancer.

The present study had several limitations. First, we detected chemokines using a cytokine/chemokine array that has limited coverage and may not capture all cytokines, secreted proteins, or exosomal interactions. Further studies are necessary to investigate the combined effects of multiple cytokines on ATCSCs. Additionally, CXCR2 and CCR6 inhibitors were utilized in our experiments. This inhibitor not only impedes the interaction between CCL20 and CCR6 but also induces alterations in downstream pathways. Further validation of the combined effects of these cytokines will be required in subsequent studies. Mechanistically, we observed upregulation of IRAK1 and p‐IRAK1 after CCR6 activation; however, the precise mechanisms underlying this upregulation remain to be elucidated. Due to the rarity of ATC cases, specimen collection was limited, and the short median survival period resulted in loss to follow‐up in some patients. The small sample size of 44 patients may have introduced statistical bias. Despite these limitations, we uncovered a novel crosstalk in the ATC. Consequently, we aimed to explore new therapeutic strategies for ATC treatment based on this crosstalk.

## 4 Conclusion

**Figure 7:**
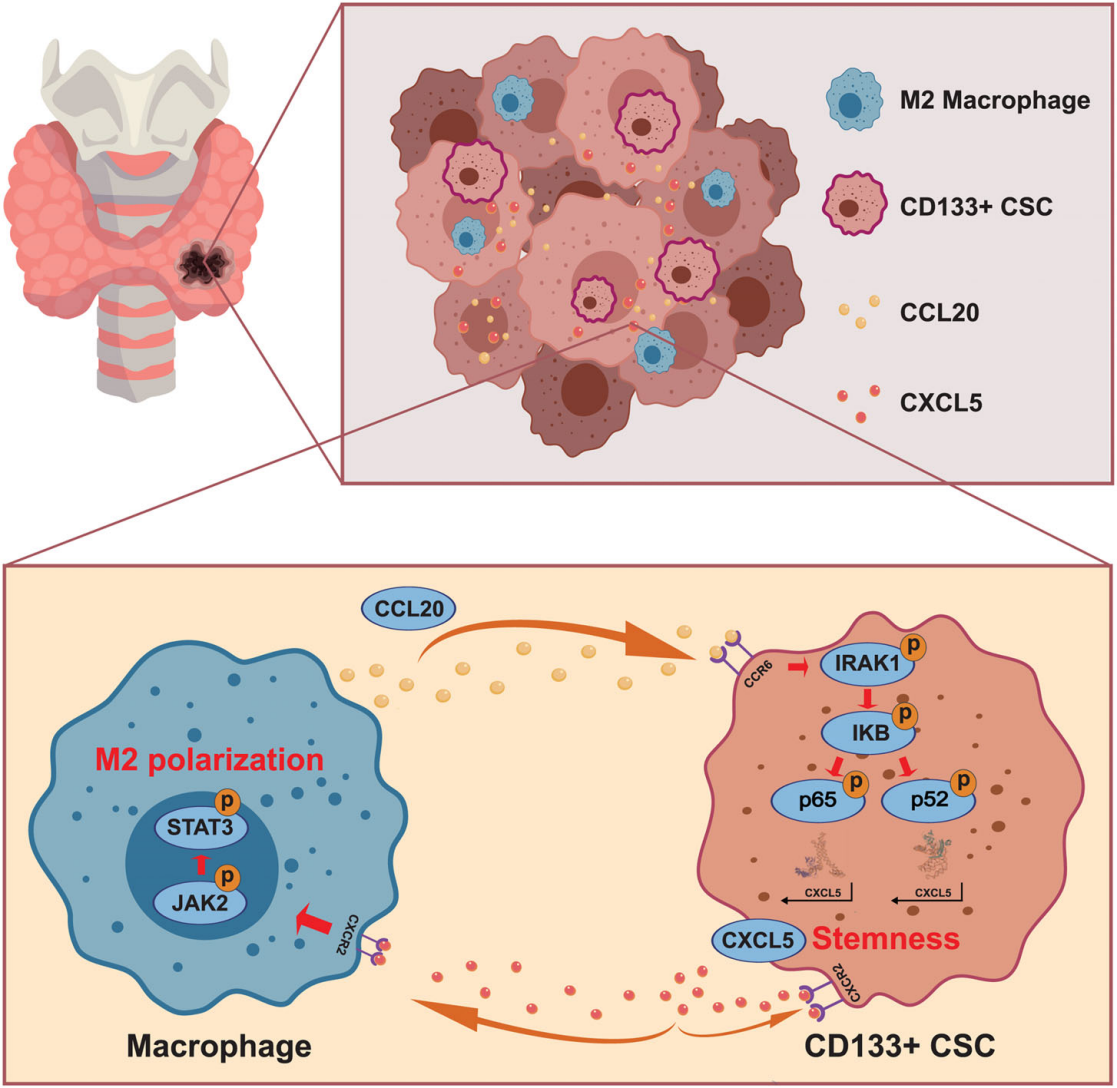
Illustration of the crosstalk between THP‐1‐M2 and ATCSC via the CCL20/CXCL5 axis.

Our study elucidates the substantial crosstalk between TAMs and ATCSCs mediated by CCL20/CXCL5. This finding is crucial for elucidating the mechanisms underlying the maintenance of stemness in ATCSCs and the development of a progressive TME (**Figure** **7**). Our research suggests that targeting CCL20/CXCL5‐mediated crosstalk could potentially serve as a promising immunotherapeutic strategy for ATC treatment.

## 5 Experimental Section

### Cell Culture

8305C, CAL62, and THP‐1 cells were cultured in MEM, DMEM, and RPMI‐1640 media, respectively, supplemented with 10% FBS (Gibco, USA). All cells were maintained in a sterile environment at 37 °C with 5% CO2. These cell lines underwent STR identification every six months.

THP‐1 cells were differentiated into adherent THP‐1‐Mφ macrophages following 24‐hour treatment with 50 ng mL^−1^ phorbol‐12‐myristate‐13‐acetate (PMA). To induce the differentiation of THP‐1‐M1 macrophages, LPS (100 ng mL^−1^) and IFN‐γ (20 ng mL^−1^) were administered for 48 h. Conversely, IL‐4 (20 ng mL^−1^) and IL‐13 (20 ng mL^−1^) were administered for 48 h to induce the differentiation of THP‐1‐M2 macrophages.

### Lentivirus Packaging, Infection, and Establishment of Stably Transfected ATC Cell Lines and ATCSCs

8305C and CAL62 cells, exhibiting a fusion rate of 30–40%, were utilized for lentiviral transduction. Lentiviral vectors were introduced into the cells at a multiplicity of infection (MOI) of 20 in accordance with the manufacturer's protocol. Furthermore, polybrene was incorporated to enhance transduction efficiency. Following 48 h of incubation, the cells underwent puromycin gradient selection to establish stably transfected cell lines. These cell lines were subsequently employed to establish ATCSCs. The lentiviral sequences are delineated in the Supplementary Materials.

### Co‐Culture System and Harvest of CM

For indirect co‐culture, THP‐1‐Mφ macrophages were placed in the upper chamber of a transwell with a pore size of 0.4 µm, while transfected ATCSCs were placed in the lower chamber. Following 48 h of co‐culture, cells were harvested for subsequent experiments.

Conditioned medium (CM) was obtained by collecting supernatants from cultured ATCSCs after centrifugation and stored for subsequent experiments. For the THP‐1‐M2 macrophage CM, the original supernatant was discarded, the cells were washed with PBS and then incubated with serum‐free 1640 medium for 24 h.

### Sphere Formation Assay

8305C and CAL62 cells were enzymatically dissociated and resuspended in DMEM/F12 medium supplemented with 1% B27 (Thermo USA), 20 µg mL^−1^ b‐FGF (PeproTech, USA), and 20 µg mL^−1^ EGF (PeproTech, USA). The cells were subsequently cultured in ultra‐low attachment culture flasks (Corning 3814) or ultra‐low attachment culture wells (Corning 3473). Following a 1‐week incubation period, the spheres were imaged, and the sphere‐forming efficiency was quantified.

### Cell proliferation and Transwell assay

Lentivirus‐transfected 8305C or CAL62 cells (3000 cells per well) were cultured in a 96‐well plate. Following cellular adherence to the wells, 10 µL CCK‐8 reagent (MCE USA) was added at 0, 24, and 48h according to the experimental protocol. The cells were subsequently incubated for an additional 2h. Absorbance was measured at 450 nm utilizing a microplate reader (Thermo Fisher Scientific). Concentration‐response curves were generated using GraphPad Prism 8.0.

The cell migration assay was conducted using a transwell (Corning 3422) with a pore size of 8 µm without Matrigel coating. The invasion assay was performed using a transwell (Corning 3422) with a pore size of 8 µm and pre‐coated with Matrigel. THP‐1‐Mφ macrophages (1 × 105) were suspended in RPMI 1640 medium supplemented with 10% FBS, and the cell suspension was introduced into the upper chamber of the Transwell. RPMI 1640 medium supplemented with 10% FBS was added to the lower chamber. Following a 48‐h incubation period, the Matrigel and residual cells in the upper chamber were removed using a cotton swab. Cells on the lower surface of the membrane were fixed with 4% methanol and stained with 0.5% crystal violet. The same methodology was applied to the lentivirus‐transfected ATC cells. Cells in randomly selected microscopic fields were counted and photographed.

### Magnetic‐Activated Cell Sorting (MACS)

The spheres were dissociated into single cells utilizing Accutase solution (Sigma, USA). Subsequently, the dissociated cells were resuspended in a solution containing BSA. All reagents and materials employed for magnetic‐activated cell sorting were obtained from Miltenyi Biotec. CD133^+^ cells were isolated using CD133/1 MicroBeads in accordance with the manufacturer's instructions. Sorting efficiency was evaluated by flow cytometry using labeled‐PE (CD133/2).

### Quantitative Reverse Transcription PCR (RT‐qPCR)

Total RNA was extracted from the cells utilizing TRIzol reagent (Invitrogen). Total RNA (1 µg) was reverse‐transcribed into cDNA employing a cDNA synthesis kit, in accordance with the manufacturer's protocol. Real‐time quantitative PCR was conducted using qMIX in a final volume of 10 µL. Alterations in expression levels were normalized utilizing the ΔΔCT method, with GAPDH serving as the control. Data analysis was performed using GraphPad Prism 8.0.

### Western Blotting

Total protein lysates (20–30 µg per lane) were separated utilizing 8–12% SDS‐PAGE and subsequently transferred onto PVDF membranes. Immunoblotting was conducted employing specific antibodies and detected using ECL substrate. The signals were detected utilizing a Bio‐Rad imaging system, and analysis was performed employing the ImageLab software. The primary antibodies utilized in this study are enumerated in the Supplementary Materials.

### Elisa Assay

The concentrations of CXCL5 and CCL20 in the supernatants of TAM and ATCSC were quantified utilizing human CXCL5 (Multisciences China) and CCL20 (Proteintech USA) ELISA kits, respectively, in accordance with the manufacturer's protocols. The absorbance was measured at 450 nm employing a microplate reader (Thermo Fisher Scientific). Cytokine concentrations were subsequently determined using GraphPad Prism 8.0.

### Flow Cytometry

Single cells were resuspended in PBS and subsequently stained with specific monoclonal antibodies anti‐CD133 in ATCSCs under dark conditions. Immunofluorescence co‐staining was conducted utilizing monoclonal antibodies antiCD86 and antiCD206 to evaluate their expression in TAMs. Antibodies, including appropriate isotype controls, were obtained from BioLegend.

### ChIP Assay

PBS and 100 mM PMSF were pre‐cooled at 4 °C, whereas the SDS Lysis Buffer was pre‐warmed at 37 °C. Subsequently, the solutions were dissolved and combined. ATCSCs were harvested by centrifugation, washed with pre‐cooled PBS containing 1 mM PMSF, and resuspended. The cells underwent a second wash and were centrifuged at 15000 × *g* for 15 min. The supernatant was collected. Immunoprecipitation was conducted using NF‐κB1 or NF‐κB2 antibodies. Incubation was carried out overnight at 4 °C. The supernatant served as the positive control, while rabbit IgG antibody served as the negative control. Protein A/G magnetic beads were combined with the antibody‐antigen complex solution and subsequently isolated. The resulting mixture was heated in proteinase K elution buffer at 65 °C for 2 h. DNA fragments were purified utilizing a commercial genomic DNA extraction kit.

### Dual‐Luciferase Reporter Assay

CAL62‐spheres and 8305C‐spheres (2 × 104 cells per well) were seeded in ultra‐low‐attachment 96‐well plates (Corning 3474). The spheres were subsequently transfected with full‐length or truncated CXCL5 promoter luciferase reporter plasmids at a final concentration of 200 ng/100 µL. Spheres were harvested 24 h post‐transfection. Luciferase activity was measured utilizing a NanoGlo Dual‐Luciferase Reporter Assay System. GloEx lysis buffer (25 µL) was added to each well, and the plate was agitated at 100 rpm for 15 min to ensure thorough mixing of the samples. GloExFluc reagent (100 µL) was added to each well and the plate was agitated for 3 min at 100 rpm to ensure thorough mixing of the samples. The luminescence intensity of firefly luciferase was quantified. NanodlrStop&Glo reagent (100 µL) was added to each well and mixed thoroughly. The plates were agitated for 3 min at 100 rpm. The luminescence intensity of Renilla luciferase was quantified. The full‐length CXCL5 promoter luciferase reporter plasmid was subjected to mutagenesis; the mutated sequences are provided in the Supplementary Materials. The experiment was replicated following mutation to determine luciferase activity.

### Immunohistochemistry and Immunofluorescence Analyses

ATC samples were obtained from the First Hospital of China Medical University. Paraffin‐embedded sections of ATC tissues were sectioned to a thickness of 4 µm utilizing a continuous cutting method. Following deparaffinization, rehydration, and antigen retrieval, endogenous peroxidase activity was quenched. The specimens were subsequently incubated with anti‐human CD133 monoclonal antibodies (1:500; Abcam, USA), CD163 (1:50; Abcam, USA), and CXCL5 (1:50; Abcam, USA) overnight at 4 °C. Immunostaining was performed using DAB in accordance with the manufacturer's instructions. Isotype‐matching antibodies served as negative controls. Immunoreactivity scores were determined by multiplying the staining intensity with the percentage of positive cancer cells. This study was approved by the Ethics Committee of the First Hospital of China Medical University and adhered to the ethical guidelines established by the Ethics Committee.

### Microarray information

The GEO (GSE33630, GSE29265, and GSE76039) datasets were obtained from GEO and comprised 65 normal thyroid tissue samples, 69 PTC samples, and 40 ATC samples. Batch effects between datasets were mitigated utilizing the “Remove batch Effect” function in the limma package while preserving the intergroup differences in the R 4.1.3 environment.

### Molecular Docking Prediction

Molecular docking was employed to validate the binding activity between protein‐protein/small molecules or protein‐nucleic acids. The HDOCK online platform (http://hdock.phys.hust.edu.cn/) was utilized as the molecular docking program in this investigation. NF‐κB1 and NFκB2 proteins with UniProt database IDs P19838 and Q00653, respectively, were obtained, and the two nucleic acid sequences were modeled utilizing the 3dRNA server. HDOCK facilitated the analysis of different conformations of protein‐nucleic acid docking, the binding activity under various conformations, as well as the amino acid residues and bases within 5 Å of interaction distance. Pymol software was employed to visualize the three‐dimensional conformation of the protein‐nucleic acid docking complexes.

### Chemokine/Cytokine Array

The CM from each group was analyzed for cytokine and chemokine levels utilizing a human XL cytokine protein array (R&D Systems, ARY022). In accordance with the manufacturer's protocol, each membrane contained duplicate spots for 102 distinct soluble human cytokines. The pixel density of each spot on the array was quantified, and the mean signal of duplicate spots was calculated.

### Quantification of CD133, CD163, and CXCL5 Staining in Tissue Sections by TissueFAX Cytometry

HistoQuest software 6.0.1.130 (TissueGnostics, Vienna, Austria) was utilized to analyze CXCL5, CD133, and CD163 staining images. Employing this software, two markers were established: hematoxylin as the “main marker” and CXCL5, CD133, and CD163 as the “non‐main markers.” The following parameters were adjusted to achieve optimal cell detection: (a) nucleus size, (b) discrimination by region, (c) identification in gray, and (d) background. Real‐time reverse gating technology was employed to visualize the corresponding cells in the region of interest and evaluate the percentage of cells exhibiting positive expression.

### Xenogeneic Orthotopic Thyroid ATCSC Transplantation Models

Female BALB/c‐nu mice, aged 4–5 weeks, were obtained from Charles River. The mice were housed in a specific pathogen‐free (SPF) environment in accordance with the guidelines of the Animal Welfare and Ethics Committee of China Medical University. Following a one‐week acclimation period, the mice were administered pentobarbital sodium (600 mg kg^−1^) intraperitoneally, followed by isoflurane inhalation for anesthesia induction. The right thyroid tissue was dissected and exposed utilizing a dissecting microscope. A suspension of Luc‐labeled 8305C‐spheres was injected into the right thyroid tissue using a 10 µL Hamilton syringe equipped with a 36G needle. Following disinfection of the subcutaneous and skin sutures, the mice were monitored closely for alterations in tumor growth, weight, and water and food intake. In the Sh‐vector+CCL20 group, CCL20 (100 ng) was administered intravenously 24 h prior to transplantation with subsequent weekly injections. Tumor growth was evaluated using a live animal imaging system after five weeks.

For the limiting dilution experiments, varying quantities of 8305C‐spheres were subcutaneously injected into BALB/c‐nu mice in each group. Tumor growth was assessed and statistically analyzed after four weeks.

### Unsupervised Clustering and Dimensional Reduction

The normalized expression profiles from all samples utilizing the “merge()” function in R v4.1.3 were merged. The “harmony” package (v0.1.0) was employed to address batch effects. Batch effect correction was conducted using the top 5000 highly variable genes (HVGs) identified by the FindVariableFeatures() function from the merged dataset. Subsequently, scaled and batch‐effect‐corrected expression profiles for all samples for downstream analyses were obtained. Following batch effect correction, the top principal components (PCs) using the gene expression profiles of the top 5000 HVGs were computed. To determine the optimal number of PCs for further analysis, the PCElbowPlot() function in Seurat, as recommended in version 4.2.0 was utilized. For cell clustering, FindNeighbors() and FindClusters() functions in Seurat were applied. To obtain appropriate visualizations, RunUMAP() functions were employed. Cell identities within each cluster based on the expression of known marker genes were defined and assigned the cell identity of each cluster based on the expression of established marker genes. In the initial round of “low‐resolution” clustering, myeloid cells (CSF1R, FCER1G, LYZ), T and NK cells (CD3D, IL7R, NKG7), B cells/plasma cells (CD79A, MS4A1, IGKC, JCHAIN), mast cells (TPSB2, TPSAB1, CPA3), progenitor cells (MKI67, STMN1), epithelial cells (TG, EPCAM, KRT19), fibroblasts (RGS5, DCN, COL1A1, ACTA2), and endothelial cells (RAMP2, VWF, and FLT1) were identified. Subsequently, the second round of “high‐resolution” clustering was conducted to identify finer subclusters within each significant cell type.

### Identification of Signature Genes for Cell Clusters

The FindAllMarkers() function in Seurat was utilized to identify differentially expressed genes (DEGs) within each sub‐cluster. The statistical significance of these genes was evaluated using the Wilcoxon rank‐sum test with the Bonferroni correction. The selection criteria for signature genes were as follows: (1) expression in >20% of cells in either or both groups, (2) |log2FC| > 0.5, and (3) adjusted *p*‐value < 0.05, for the Wilcoxon rank‐sum test.

### Cell‐Cell Communication Analysis

To evaluate interaction frequencies and intensities and investigate potential communication between distinct cell types based on the expression of ligand‐receptor pairs, the “CellChat” package (version 1.5.0) with default parameters was utilized. The inclusion of a receptor or ligand in the subsequent analysis necessitated expression in >10% of cells within a cluster. Empirical *p*‐values were calculated by randomly assigning cluster labels 1000 times for each cell pair to determine the statistical significance of a ligand‐receptor pair between the two clusters.

### Statistical Analysis

Statistical analyses were performed using GraphPad Prism 8. Results are presented as the mean ± standard deviation (SD) for at least three experiments. Student's *t*‐test was used to compare differences between the two groups with a normal distribution. In cases of unequal variance among groups, analysis was performed using the Wilcoxon or Welch's *t*‐test. One‐way analysis of variance (ANOVA) or two‐way ANOVA was used to analyze data with normal distribution involving three or more groups. Mann–Whitney U analysis was used to compare mRNA expressions in thyroid cancer patients from the GEO or TCGA database. The correlation between CD133^+^ CSC and CD206^+^ M2 macrophages was analyzed using a Pearson correlation analysis. Statistical significance was set at *p* < 0.05.

### Ethical Approval and Consent to Participate

This study was approved by the Ethics Committee of the First Hospital of China Medical University ([2023]‐36). All animal experiments were approved by the Laboratory Animal Welfare and Ethical Committee of China Medical University (CMUXN2022079).

## Conflict of Interest

The authors declare no conflict of interest.

## Author Contributions

H.Z. and Y.Y. contributed equally to this work. Z.H. and Y.Y.Y. designed the study; L.Q., W.Y., S.M.Y., and S.J.Y. conducted the experiments; Z.P. and S.W. collected the clinical samples and C.Y.A. and J.X.Y. provided technical support for the experiments; H.J.P. and M.B. performed the statistical analyses; L.Q. and S.J.Y. wrote the manuscript; S.J.Y. and S.W. edited the manuscript; and all authors reviewed and approved the manuscript.

## Supporting information



Supporting Information

Supplemental Table 1

## Data Availability

Mass spectrometry proteomics data were deposited in the ProteomeXchange Consortium (http://proteomecentral.proteomexchange.org) via the iProX partner repository with the dataset identifier PXD046889.
