# Supplementary material for: CCL20/CXCL5 Drives Crosstalk Between Anaplastic Thyroid Cancer Stem Cells and Tumor‐Associated Macrophages to Promote Tumor Progression
Source: Adv Sci (Weinh). 2025 Mar 16;12(17):2405399. doi: 10.1002/advs.202405399 (PMC12061268; doi:10.1002/advs.202405399)
Supplement: Supplementary file 1 — Supporting Information [file ADVS-12-2405399-s002.docx]

Supporting Information

**
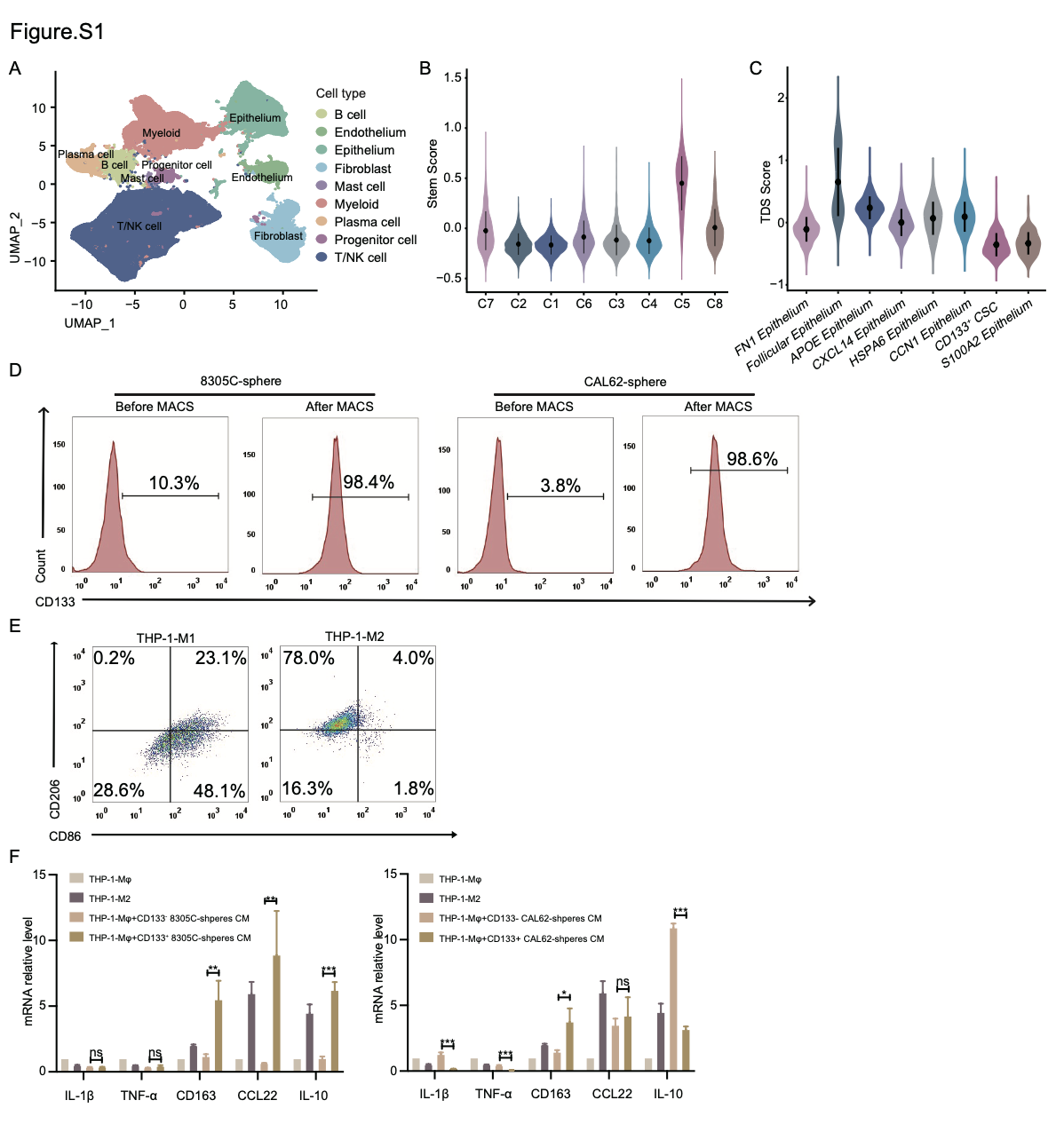
**

**Figure S1**

**A.** UMAP of all cells post-quality control and filtering grouped by the major cell type. **B.** Stemness score of various epithelial cell clusters. **C.** Thyroid differentiation score of various epithelial cell types**. D.** Flow cytometric analysis of changes in CD133 positive rates before and after magnetic-activated cell sorting of spheres (n=3). **E.** Flow cytometry analysis of changes in CD206 or CD86 positive rates of THP-1-M1 and THP-1-M2 (n = 3). **F.** The mRNA levels of IL-1β, TNF-α, CD163, CCL22, and IL-10 were measured in THP-1-Mφ, THP-1-M2, and THP-1-Mφ+CD133^+/-^ sphere CM (n = 3). *P < 0.05, **P < 0.01, ***P < 0.001, ns, no significance; Student’s t test, Two-way ANOVA test, Error bars, mean ± SD.

**Figure S2**

**A.** The mRNA levels of IL-24, ICAM-1 and MIF were compared in normal thyroid tissue, PCT and ATC from GEO datasets. **B.** The mRNA levels of CXCL5 were compared in the T stage and AJCC stage from TCGA datasets. **C.** Differences in the secretion of TNF-α, MMP-9, and CCL20 between 8305C-ATCSC and THP-1-M2 macrophages were compared using a chemokine array. **D.** Correlation analysis of MMP-9 and TNF-α mRNA expression and thyroid differentiation score in human thyroid cancers from GEO datasets. **E.** Analysis of CCL20 secretion levels in THP-1-Mφ, THP-1-M1, and THP-1-M2 cells by ELISA (n=3). **F.** Protein association network analysis of CXCL5 and CCL20 using STRING. **G.** Analysis of CXCL5 secretion levels in ATCSC stimulated by MMP-9, TNF-α, and CCL20 by ELISA (n=3). **H.** Analysis of CCL20 secretion levels in THP-1-Mφ stimulated by CXCL5 or CCL20 silencing using ELISA (n=3). **I.** Flow cytometry analysis of changes in CD206 positive rates of THP-1-Mφ under the stimulation of CXCL5 or CCL20 silencing (n=3). *P < 0.05, **P < 0.01, ***P < 0.001, ns, no significance; Mann–Whitney U analysis, Two-way ANOVA test, Error bars, mean ± SD.

**Figure S3**

**A.** GO analysis was performed on the differentially expressed proteins between the CAL62-ATCSC treated with CCL20 and the control group. **B.** The changes in luciferase activity of the NF-κB1, NF-κB2, and NF-κB1+NF-κB2 plasmids were analysed separately using dual-luciferase reporter assays (n=3). **C.** ChIP analysis of the binding ability between NF-κB1/2 and the binding elements (n=3). **D.** Changes in the luciferase activity of NF-κB1/2 and its mutated forms were analysed using dual-luciferase reporter assays (n=3). **E.** ChIP analysis of the binding ability between NF-κB1/2 and the binding elements after CCR6 inhibition (n=3). **F.** Analysis of CXCL5 secretion levels in ATCSC under the stimulation of CCL20 or CCR6 inhibitor by ELISA (n=3). *P < 0.05, **P < 0.01, ***P < 0.001, ns, no significance; Student’s t test, Two-way ANOVA test, Error bars, mean ± SD.


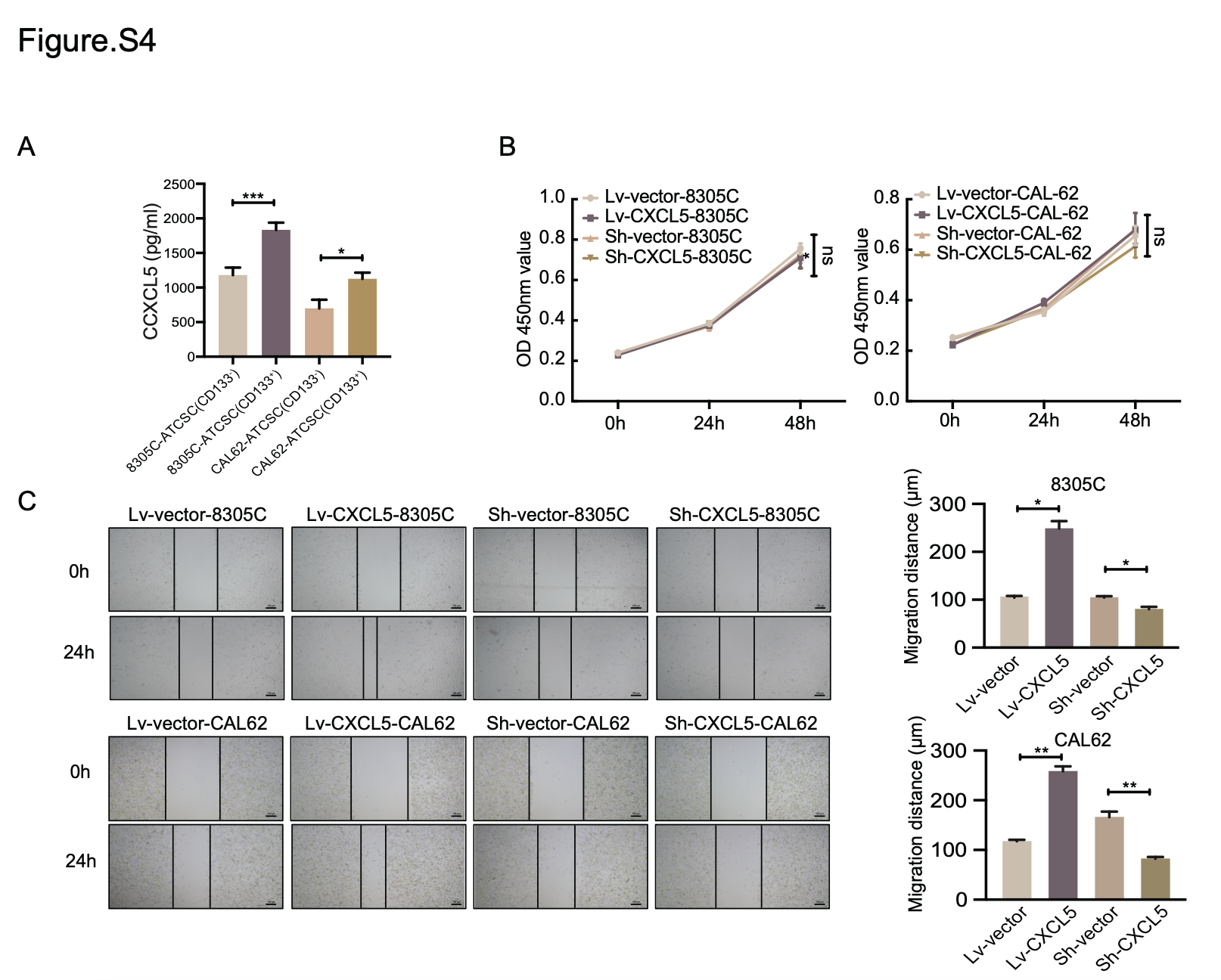


**Figure S4.**

**A.** Analysis of CXCL5 secretion levels in CD133+ or CD133- ATCSC (n=3). **B.** CCK8 was used to analyze the difference in the proliferation of 8305C and CAL62 between CXCL5 overexpression or silencing (n=3). **C.** Changes in the migration ability of 8305C and CAL62 overexpression or silencing of CXCL5 were analysed by wound healing assay (n=3). *P < 0.05, **P < 0.01, ***P < 0.001, ns, no significance; Student’s t test, Two-way ANOVA test, Error bars, mean ± SD.
